# Spatiotemporal Regulation of Hierarchical Assembly of Hydroxyapatite for Enamel Remineralization

**DOI:** 10.1002/advs.78180

**Published:** 2026-10-08

**Authors:** Zhenhang Tang, Zhisen Zhang, Yuanyu Zhou, Wenzhi Wu, Congrui Zhao, Chang Liu, Tong Shi, Wenjing Jin, Zhaoming Liu, Weifeng Fang, Qingyun Lin, Yi He, Zhijian Xie, Ruikang Tang, Changyu Shao, Zhuo Chen

**Affiliations:** ^1^ Stomatology Hospital, School of Stomatology, Zhejiang University School of Medicine Zhejiang Provincial Clinical Research Center for Oral Diseases Zhejiang Key Laboratory of Oral Biomedical, Zhejiang‐Singapore International Joint Laboratory of Oral Bioengineering, Zhejiang Clinovation Pride Engineering Research Center of Oral Biomaterials and Devices of Zhejiang Province Hangzhou China; ^2^ Department of Physics Research Institute for Biomimetics and Soft Matter Jiujiang Research Institute and Fujian Provincial Key Laboratory for Soft Functional Materials Research Xiamen University Xiamen China; ^3^ Department of Chemistry Zhejiang University Hangzhou China; ^4^ Center of Electron Microscopy State Key Laboratory of Silicon and Advanced Semiconductor Materials School of Materials Science and Engineering Zhejiang University Hangzhou China; ^5^ College of Chemical and Biological Engineering Zhejiang University Hangzhou China

**Keywords:** enamel remineralization, enzymatic mineralization system, hierarchical assembly, hydroxyapatite, interfacial energy modulation

## Abstract

The hierarchical structure of tooth enamel confers exceptional mechanical robustness, yet its biomimetic regeneration remains a significant challenge. We develop an enzymatic mineralization system (EMS) that achieves enamel regeneration by directing the hierarchical assembly of hydroxyapatite (HAP). Within the EMS, the alkaline phosphatase hydrolyzes β‐glycerophosphate (β‐GP), triggering the mineralization reaction of calcium phosphate (CaP) and controlling its structural evolution. The mineralization process is characterized by a series of spatiotemporal events, in which CaP prenucleation clusters initially form and transform into HAP nanorod building blocks; these units then hierarchically self‐assemble into microscale crystalline bundles that resemble native enamel rods. Mechanistically, differential adsorption of β‐GP on the (100) and (002) facets of HAP, together with facet‐dependent enzymatic cleavage of surface‐bound β‐GP, generates high‐energy facets and interfacial energy differences that drive the hierarchical assembly, beginning with *c*‐axis–oriented attachment followed by lateral aggregation. Extending this new strategy to enamel remineralization, the EMS restores the native microstructure and essential mechanical properties. Moreover, a hydrogel‐based EMS demonstrates clinical translatability through effective in vivo repair. This paradigm of interfacial energy–modulated assembly provides a robust blueprint for the bottom‐up construction of complex, high‐performance biomimetic materials.

## Graphical abstract

Biomimetic repair exhibits unique advantages for enamel regeneration compared with conventional clinical treatments. An enzymatic mineralization system (EMS) is developed, where the resulting HAP nanorods hierarchically assemble into a highly ordered structure on the enamel surface through the spatiotemporal modulation of interfacial energies of crystal facets. As a result, enamel is reconstructed with its native microstructure and mechanical strength.

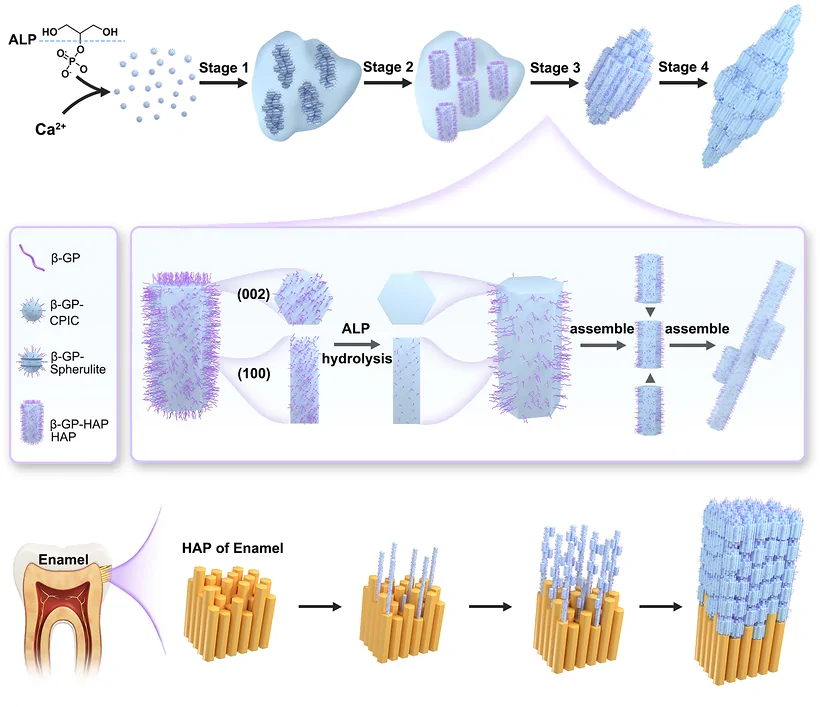

## 1 Introduction

Tooth enamel, an outstanding natural biomaterial, is primarily composed of hydroxyapatite (HAP) crystals, hundreds of micrometers in length and 20–50 nm in width. These crystals serve as its fundamental building blocks and are densely packed together, forming the rods and inter‐rods [1]. The inter‐rod surrounds each rod at an angle of approximately 60° and, together, they assemble into a higher‐order hierarchical structure that endows enamel with robust mechanical properties and exceptional wear resistance [2]. Nevertheless, despite its mechanical robustness, enamel remains susceptible to chemical attack, primarily through the acids produced by cariogenic bacteria, leading to the onset of dental caries. As an acellular tissue, enamel is incapable of self‐repair when damaged [3]. Inspired by biomineralization, a wide range of strategies have been explored to remineralize demineralized enamel. These approaches range from the regulation of HAP nucleation and growth, mediated by organic additives such as amelogenin [4] and its mimics [5, 6, 7], and elastin‐like recombinamers [8, 9], to the use of biomimetic mineral precursors, where organic stabilization enables control over the amorphous‐to‐crystalline transformation [10, 11]. However, these strategies are constrained by several limitations, including the use of toxic reagents (e.g., triethylamine), an inability to replicate the hierarchical architecture of natural enamel, and poorly controlled mineralization processes, all of which significantly impede their further application. Therefore, developing advanced strategies capable of reconstructing the hierarchical structure of enamel and restoring its mechanical functionality via biosafe, bio‐inspired pathways remains a critical challenge.

Enamel formation (amelogenesis) is a multi‐stage process orchestrated by sequential molecular pathways in ameloblasts, the enamel‐forming cells [12]. During this process, amelogenin co‐assembles with calcium phosphate (CaP) particles to form linear chains, which then organize into parallel arrays and ultimately fuse into bundles of elongated crystallites [13]. This structural maturation may be facilitated by enzymatic processing, wherein the controlled degradation of protein promotes crystal growth (such as the amelogenin processed by matrix metallopeptidase 20) [14, 15]. In addition, numerous studies have shown that alkaline phosphatase (ALP) not only supplies orthophosphate ions via the hydrolysis of phosphomonoesters but also participates in the entire process of enamel formation [16, 17, 18]. These findings underscore that amelogenesis depends on the guided assembly of mineral particles and highlight the essential contribution of spatiotemporal regulation, which is mediated by enzymatic control, in biomineralization. However, the molecular mechanisms by which organic molecules, particularly enzymes and proteins, regulate mineral particle assembly during mineralization remain largely unknown.

Given the central roles of CaP particle assembly and enzymatic hydrolysis in amelogenesis, here, we have developed a biomimetic enzymatic mineralization system (EMS). In this system, ALP hydrolyzes β‐glycerophosphate (β‐GP) to release phosphate ions, which subsequently react with calcium ions to form CaP minerals. More critically, a sequential biomimetic process is initiated by ALP‐catalyzed hydrolysis of β‐GP at CaP particle surfaces. The initial hydrolysis event destabilizes CaP clusters, inducing their aggregation into a continuous amorphous calcium phosphate (ACP), within which HAP progressively nucleates and grows into nanorods (Stage 1 and 2; Figure 1A). Subsequently, these nanorods, serving as the fundamental building blocks, undergo hierarchical assembly driven by interfacial energy modulation arising from localized interfacial enzymatic reactions (Stage 2 and 3; Figure 1A). This process ultimately culminates in the coalescence of these assemblies into HAP bundles that closely mimic natural enamel rods (Stage 4; Figure 1A). On the basis of these mechanistic insights, we have established an EMS‐based platform for enamel remineralization that replicates the complex architecture of enamel, thereby restoring its intrinsic mechanical robustness and tribological performance. The therapeutic efficacy of this strategy in an in vivo model underlines its translational potential for the treatment of early‐stage dental caries. Beyond dental enamel repair, this synthetic strategy enables the ordered assembly of inorganic particles from fundamental nanoscale building units into microscale structural materials by dynamically modulating interfacial energies through facet‐selective enzymatic hydrolysis, providing a versatile blueprint for engineering functional materials with long‐range‐ordered structures and exceptional mechanical performance.

**FIGURE 1:**
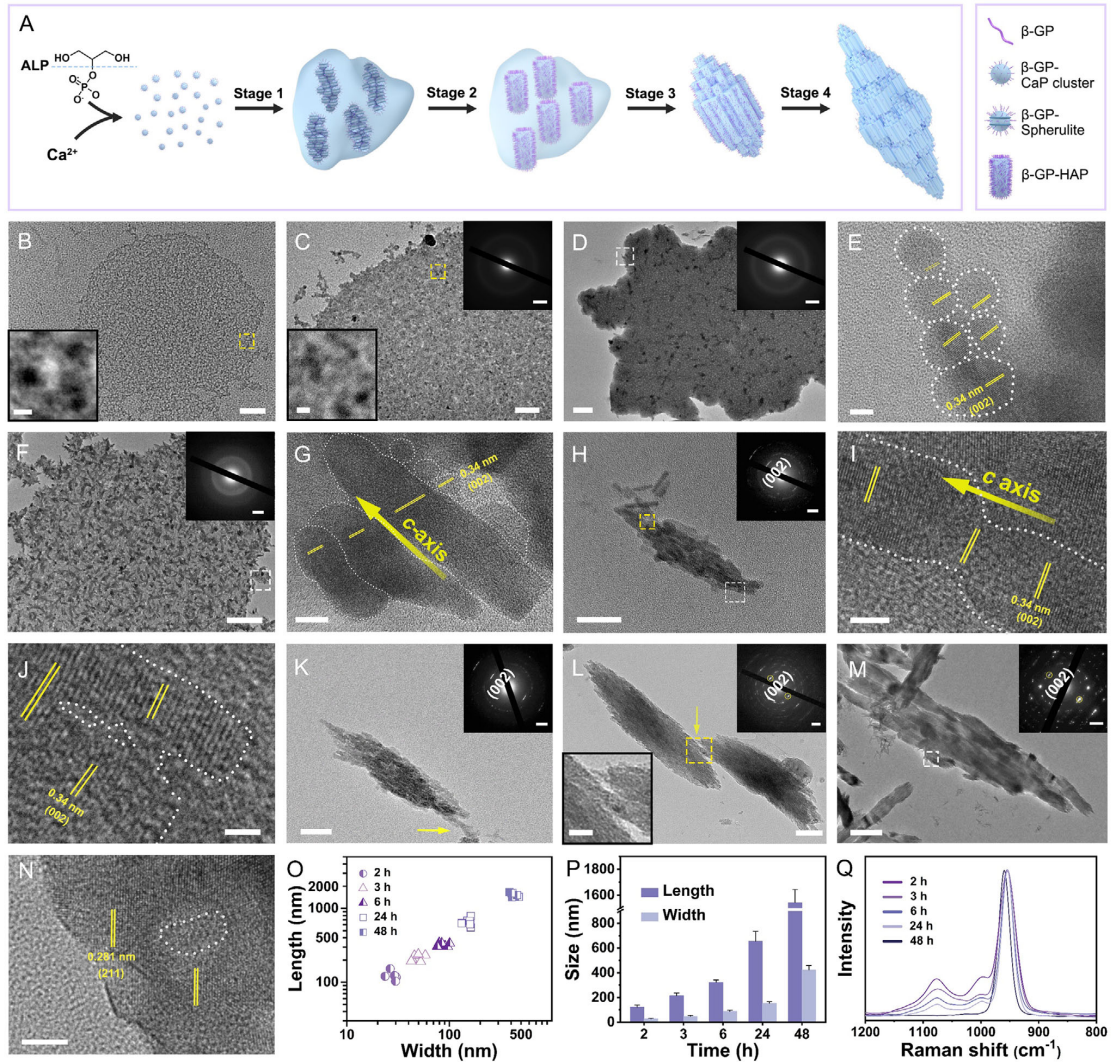
Hierarchical assembly of CaP particles. (A) Schematic illustration of the entire EMS process was created using Blender (Blender Foundation, Amsterdam, the Netherlands). (B) CaP clusters with a diameter of approximately 2 nm were observed within the EMS after 2 min. (C) CaP clusters aggregate into a continuous ACP after 30 min. (D) After 1 h, ACP transformed into poorly crystalline HAP nanocrystals. (E) HRTEM image corresponding to the region within the white square in (D) shows the spherical HAP nanocrystals assembling and fusing. (F) After 2 h, the HAP nanorods formed and began to assemble with a preferred orientation. (G) The HRTEM image displays the area within the white square in (F). (H‐J) After 3 h, spindle‐shaped HAP mesocrystals composed of aligned HAP nanorods were formed. The HRTEM image of the region within the white and yellow squares in (H) reveals that HAP nanorods can coalesce along both the *c*‐axis (I) and the *a/b*‐axis (J). (K) After 6 h, larger spindle‐shaped HAP mesocrystals formed through the assembly of HAP nanorods. (L) After 24 h, two larger HAP mesocrystals coalesced into a single entity, bridged by a dividual HAP nanorod (indicated by the yellow arrow and shown enlarged at bottom left). (M) After 48 h, HAP mesocrystals assembled to form crystal bundles. (N) HRTEM image displays the area indicated by the white square in (M). (O) The size of HAP nanorods changes over time. (P) The length and width of HAP nanorods maintain a constant aspect ratio over time. (Q) Raman spectra of the CaP minerals acquired at various time points. Upper‐right insets in (C), (D), (F), (H), (K), (L), and (M) show SAED patterns: the absence of diffraction spots or rings indicates an amorphous state, whereas the presence of arcs or spots corresponds to crystalline HAP. Lower‐left insets in (B), (C), and (L) display the corresponding high‐magnification TEM images of the regions within the yellow squares. Scale bars: 50 nm (B, C, K); 200 nm (D, M); 5 nm (E, I, N); 500 nm (F); 10 nm (G); 20 nm (H); 2 nm (J); 100 nm (L); 1 nm^−1^ (upper right insets in C, D, H and K); 2 nm^−1^ (upper right insets in F, L and M); 2 nm (lower left inset in B); 5 nm (lower left insets in C); 30 nm (lower left insets in L).

## 2 Results and Discussion

### 2.1 Hierarchical Assembly of Calcium Phosphate Particles

During enamel development, ALP plays a crucial role in regulating the formation and structural organization of CaP minerals (Figure S1) [16, 17, 18]. Inspired by this biological mechanism, we established an EMS in which ALP hydrolyzes β‐GP to generate phosphate ions (Figure S2). To track the temporal evolution of CaP particles, we collected samples at specific time points for transmission electron microscopy (TEM) analysis. At the very earliest stage (within 2 min), CaP clusters approximately 2 nm in diameter were observed (Figure 1B; Figure S3). These clusters were likely stabilized by the β‐GP through coordination with calcium ions (Figures S3–S5). Upon destabilization of CaP clusters, likely caused by the removal of β‐GP via ALP hydrolysis, they readily aggregated into continuous bulk ACP owing to the high aggregation propensity of ultrasmall clusters [19, 20] (Figure 1C; Figures S4, S5). After 1 h of mineralization, the ACP transformed into poorly crystalline HAP crystals with a spherical structure and exhibited a preferential *c*‐axis alignment, a phenomenon governed by interfacial energy (Figure 1D,E; Figure S6) [21]. As enzymatic hydrolysis of β‐GP proceeded both on the CaP particles surface and in solution, HAP nanorods formed and assembled with *c*‐axis alignment after 2 h of mineralization (Figure 1F,G; Figure S6). After an additional 1 h, ACP was largely consumed and predominantly converted into HAP, accompanied by the disappearance of the continuous bulk phase observed in Figure 1C‐F and the formation of spindle‐shaped HAP mesocrystals. Within and adjacent to these mesocrystals, individual HAP nanorods (26.0 ± 10.0 nm in length and 6.3 ± 0.8 nm in width) remained clearly discernible, strongly suggesting that the β‐GP‐modified HAP nanorods act as building blocks for crystal growth through a self‐assembly, as previously observed in mesocrystal systems (Figure 1H; Figure S7) [22]. Moreover, high‐resolution TEM (HRTEM) images and selected‐area electron diffraction (SAED) patterns further revealed that the HAP spindles comprise assemblages of crystallographically aligned nanorods elongated along the *c*‐axis (Figure 1I,J). These self‐assembled HAP nanorods appear to coalesce and eliminate grain boundaries, possibly through the removal of surface‐adsorbed β‐GP by ALP hydrolysis, as suggested by the nanometre‐scale pores observed in Figure 1J. Prolonging the reaction to 6 h resulted in the formation of larger spindle‐shaped mesocrystals, which grew via the oriented assembly of HAP nanorods, as indicated by a tip nanorod attaching to the mesocrystal (yellow arrow), and exhibited higher‐order alignment (Figure 1K).

Extending the reaction to 24 h not only promoted the growth of single HAP mesocrystals but also enabled the fusion of two adjacent mesocrystals via oriented attachment (OA) along a common crystallographic axis, eliminating grain boundaries and forming a larger, coherently aligned crystal (Figure 1L) [23]. At the end of the mineralization (48 h), the mesocrystals further assembled and coalesced into micro‐sized crystal bundles resembling enamel rods (Figure 1M; Figure S8). Furthermore, the HRTEM image reveals amorphous regions within the assemblies, which may originate from residual β‐GP, indicating that growth through β‐GP‐modified nanorod assembly (Figure 1N). It should be noted that this residual organic matter can enhance the mechanical performance of the resulting materials, particularly their fracture toughness, which is a strategy widely employed in natural tooth formation [24, 25]. Additionally, the corresponding SAED patterns (upper‐right inset in Figure 1L,M) display sharp, distinct diffraction spots, confirming their high crystallinity.

Dimensional analysis of the spindles showed that they began to form at 2 h, developed into complete spindle‐shaped structures by 3 h, and subsequently grew through the hierarchical assembly of CaP particles, including primary HAP nanorods and spindles. A plot of spindle length vs. width revealed a constant aspect ratio of about 4 across all HAP spindles, highlighting the deterministic nature of their growth. This consistency was maintained, with both the average spindle length and width increasing until the end (Figure 1O,P). Analysis also reveals that the entire assembly process proceeds through three distinct stages: in the first stage, the primary assembly units (HAP nanorods) form; in the second stage, spindle‐shaped HAP mesocrystals develop through the assembly of these nanorods; and in the final stage, crystal bundles form through the assembly of both spindles and nanorods. Furthermore, two control experiments were performed. In the first, mineralization was conducted in a supersaturated calcium phosphate system in the absence of ALP and β‐GP, where crystallization occurs via a classical nucleation pathway (Figure S9). In the second, mineralization was carried out in EMS without fluoride ions, under which conditions no ordered assembly was observed (Figure S10). Overall, these results suggest that enzymatic hydrolysis is the dominant factor governing the multistep hierarchical assembly process, whereas fluoride ions promote nanorod formation but do not mediate the assembly process.

Furthermore, sequential Raman spectra of the harvested CaP particles at different mineralization stages revealed a blue‐shift of the phosphate P‐O symmetric stretching peak (900‐960 cm^−1^), reflecting the enhancement of the mineral crystallinity (Figure 1Q). Concurrently, the characteristic symmetric stretching vibrations of β‐GP (P‐O at 980 cm^−1^ and P = O at 1058 cm^−1^) persisted but underwent progressive blue shifts and marked attenuation (Figure S4). These spectral evolutions indicate that β‐GP molecules actively participate throughout the entire assembly process, undergoing interfacial adsorption and continuous ALP‐mediated hydrolysis at the evolving mineral interface, which is further corroborated by the solid‐state nuclear magnetic resonance (ssNMR) analysis (Figure S11). These results further hint that the β‐GP plays a crucial role, potentially driving the assembly of CaP particles.

### 2.2 Enzymatic Hydrolysis of β‐GP Adsorbed on HAP by ALP

TEM observations, Raman spectra, and ^13^C ssNMR indicated that β‐GP can adsorb onto HAP crystals and regulate the hierarchical assembly of HAP nanorods to form an enamel‐like structure crystalline bundle. To determine whether ALP directly mediates the localized hydrolysis of β‐GP at the HAP surface, we employed Cy3‐labeled β‐GP (Cy3‐β‐GP) and Cy5‐tagged ALP (Cy5‐ALP) for confocal laser scanning microscopy (CLSM). This approach enabled spatially resolved tracking of Cy3‐β‐GP adsorption onto HAP and its subsequent enzymatic cleavage by Cy5‐ALP in situ (Figure 2A). Although Cy3 and Cy5 fluorophores are covalently linked to β‐GP and ALP, respectively, their functions remain unaffected; notably, Cy5‐ALP retains its ability to efficiently catalyze the hydrolysis of Cy3‐β‐GP (Figure S12). To visualize this interfacial process, Calcein‐labeled HAP crystals (green) were immobilized within a 3D μ‐Slide. Upon introducing Cy3‐β‐GP from the flanking channel, we observed rapid adsorption of the substrate onto the HAP surface, as evidenced by intense red fluorescence (Figure 2B). Subsequent lateral injection of Cy5‐ALP triggered a pronounced attenuation of this red signal within 5 min, indicating efficient enzymatic processing of the surface‐bound substrate (Figure 2B–E). This interfacial enzymatic reaction was further quantified via dual‐color fluorescence cross‐correlation spectroscopy (FCCS). Initially, a high cross‐correlation (CC) amplitude confirmed the stable recruitment of Cy3‐β‐GP onto the HAP surface via electrostatic interaction between phosphate moieties and calcium ions (Figure 2F). The subsequent addition of Cy5‐ALP induced the catalytic conversion of β‐GP into Cy3‐glycerol; the latter, possessing diminished interfacial affinity for the HAP crystals, desorbed into the bulk solution, thereby resulting in a marked reduction in CC amplitude. These findings collectively establish that ALP‐mediated hydrolysis governs the fate of β‐GP both in the solution and upon mineral adsorption. This mechanism likely dictates the removal of the organic substrate, driving the oriented self‐assembly of HAP nanorods into the hierarchical architectures shown in Figure 1.

**FIGURE 2:**
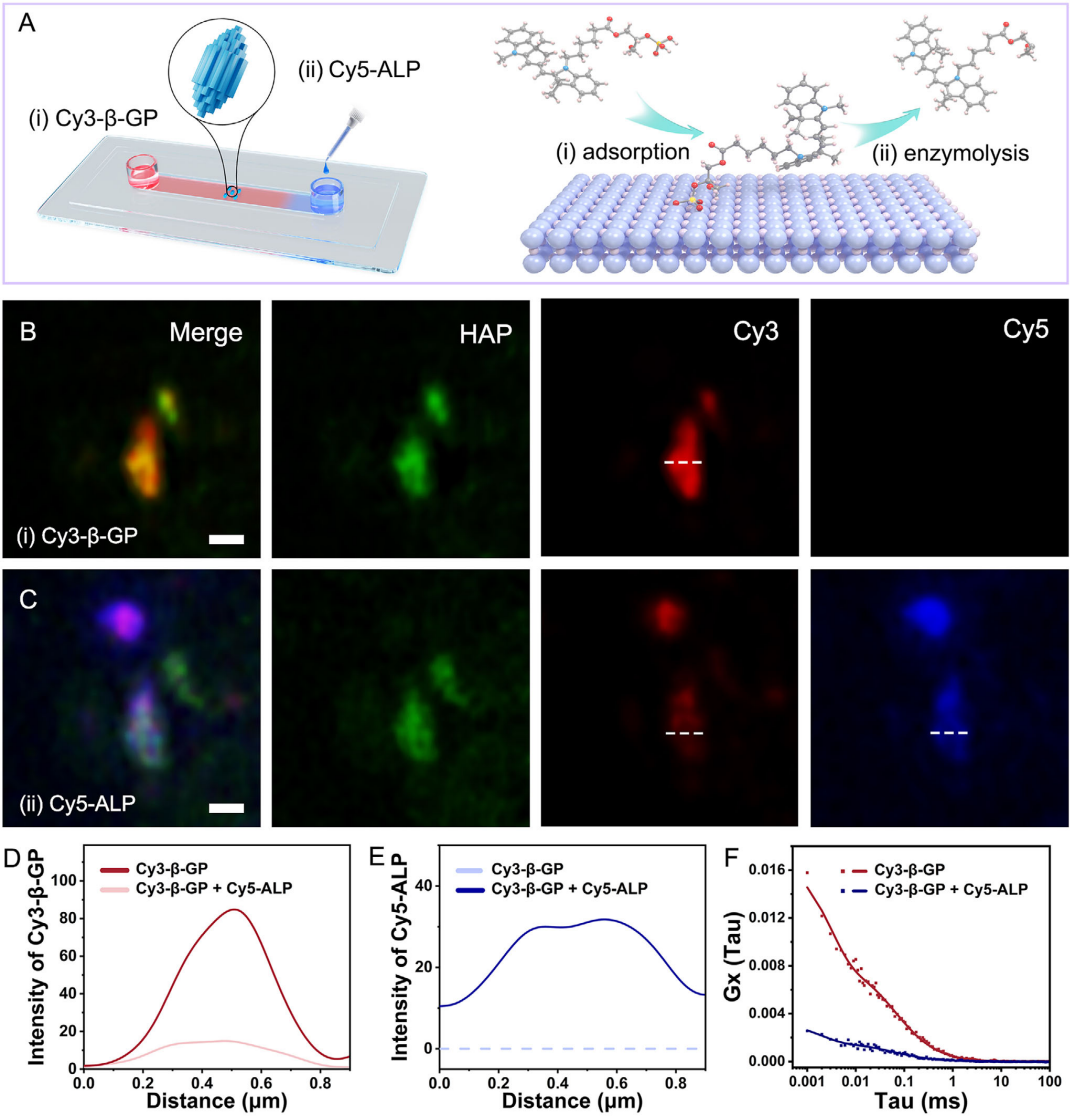
Enzymatic hydrolysis of surface‐bound β‐GP on HAP. (A) Schematic of enzymatic hydrolysis: (i) Cy3‐β‐GP adsorption onto HAP; (ii) Cy5‐ALP enzymatic hydrolysis of Cy3‐β‐GP. This schematic illustration was created using Blender. (B) Spindle‐shaped HAP crystal bundles were labeled with calcein, yielding green fluorescence, while Cy3‐β‐GP adsorbed onto HAP emitted red fluorescence. The merged green and red fluorescence appears as yellow. Scale bar: 500 nm. (C) Upon introduction of Cy5‐ALP, the red fluorescence was attenuated, indicating enzymatic hydrolysis of Cy3‐β‐GP. Subsequently, the newly bound Cy5‐ALP (blue) colocalized with HAP bundles, confirming that hydrolysis occurred at the crystal surface. Scale bar: 500 nm. (D) Intensity profiles along HAP bundles (dashed lines in B and C) demonstrate a significant decrease in the Cy3‐β‐GP signal as a result of enzymatic hydrolysis. (E) Concurrently, the emergence and intensification of the Cy5‐ALP signal confirm the enzyme adsorption onto the crystal surface. (F) FCCS analysis reveals a progressive attenuation in cross‐correlation amplitude following the addition of Cy5‐ALP, providing direct kinetic evidence for the localized hydrolysis of surface‐adsorbed Cy3‐β‐GP by the enzyme.

### 2.3 Mechanism of Hierarchical Assembly of HAP Nanorod

To elucidate the mechanism governing the hierarchical assembly of HAP nanorods, we investigated the site‐specific adsorption of β‐GP onto the HAP (100) and (002) facets as representative model surfaces based on previous studies [26, 27]. Millimeter‐sized HAP crystals with well‐defined facets were utilized to facilitate visualization (See Figure S13 for details). CLSM imaging revealed that Cy3‐β‐GP successfully localized onto both facets; however, a significantly higher fluorescence intensity on the (100) facet indicated a superior binding affinity compared to the (002) facet (Figure 3A,C,I). This facet‐specific preference was further corroborated by molecular dynamics (MD) simulations, which revealed that the adsorption density of β‐GP on the (100) facet is more than twofold higher than on the (002) facet. In addition, the phosphate groups of β‐GP were found to engage more extensively with the (100) surface through multidentate coordination (Figure 3E–H). This interaction is characterized by more numerous and stable contacts with lattice calcium ions, as quantified by a significantly higher average coordination number (CN) between β‐GP molecules and Ca^2+^ (CN = 2.90 ± 0.20 for (100) vs. 1.47 ± 0.17 for (002); Figure 3J). Furthermore, the first‐neighbor phosphate oxygen‐calcium ion (O‐Ca^2+^) radial distribution function (RDF) exhibits a sharper and more intense peak at 2.38 Å for the (100) face, with a *g*(*r*) value of approximately 3.93 compared to a significantly lower value of 1.46 for the (002) face (Figure S14). This specific distance (∼2.38 Å) aligns with typical inner‐sphere coordination between phosphate oxygen and surface calcium ions [28], indicating that β‐GP forms stronger electrostatic interactions and more stable binding configurations on the (100) crystal planes. Consequently, the adsorption energy of the β‐GP molecule is significantly more exergonic on the (100) facet (−563.05 ± 67.06 kJ/mol) than on the (002) facet (−404.95 ± 27.60 kJ/mol; Figure 3K). This energetic disparity demonstrates that the robust local ion coordination on the (100) plane translates directly into a thermodynamically superior adsorption state, providing a robust physical basis for the higher molecular surface coverage observed experimentally.

**FIGURE 3:**
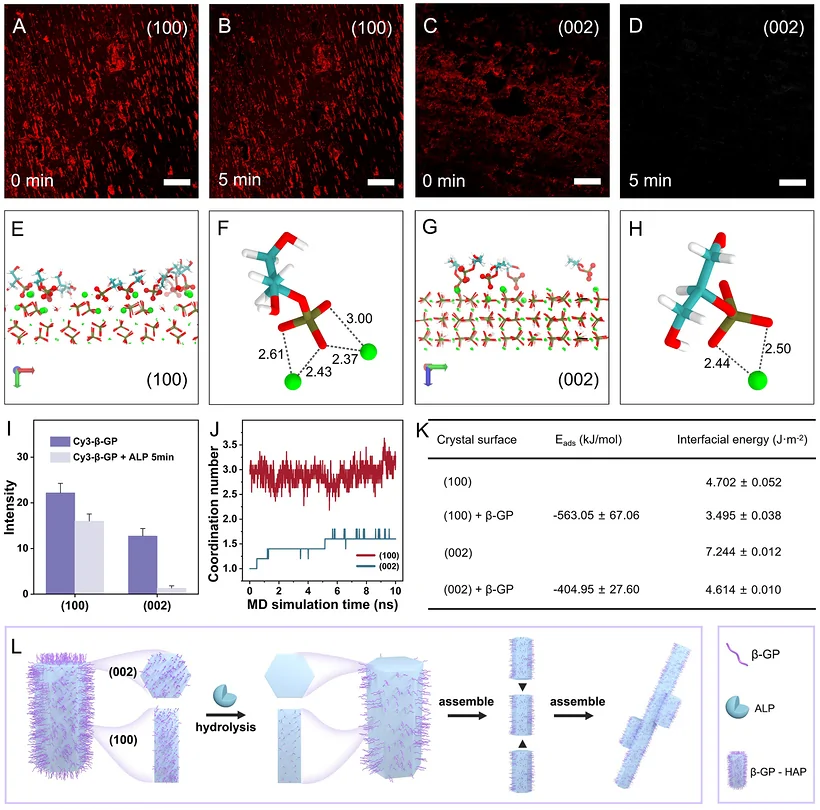
Facet‐dependent adsorption of β‐GP and its differential hydrolysis on HAP surfaces. (A‐D) In situ real‐time CLSM tracking of Cy3‐β‐GP (red) within the same region of interest (ROI) on the HAP (100) (A, B) and (002) (C, D) facets. Images were sequentially acquired from the identical field of view before (A, C) and after 5 min of localized ALP hydrolysis (B, D), enabling direct comparison of the changes in fluorescence intensity induced by ALP hydrolysis. (E, G) Representative MD simulation snapshot illustrating the equilibrium adsorption configurations of β‐GP molecules on the (100) and (002) facets. The (100) facet exhibits a significantly higher adsorption density with 11 β‐GP molecules, compared to 5 β‐GP molecules on the (002) facet. (F, H) Coordination chemistry analysis revealing distinct binding motifs: on the (100) facet, β‐GP molecules bridge adjacent lattice calcium ions through multiple Ca‐O bonds, whereas a monodentate‐like coordination to a single calcium ion predominates on the (002) facet. (Color scheme: Ca, green; O, red; H, white; C, blue; P, brown). (I) Semiquantitative fluorescence analysis showed a marginal 21% reduction in signal intensity on the (100) facet, compared to a significant 89% decrease on the (002) facet after hydrolysis. (J) Time‐resolved coordination numbers between β‐GP phosphate oxygen atoms and surface calcium ions on the (100) and (002) planes. (K) MD‐calculated adsorption energies (*E_ads_*) and the resultant facet‐specific interfacial energies, highlighting the energetic basis for controlled mineral assembly. (L) Schematic illustration of the spatiotemporally regulated sequential hierarchical assembly process, created using Blender. Scale bars, 500 nm (A‐D).

This facet‐dependent adsorption strength dictates the subsequent enzymatic processing kinetics. In situ ALP hydrolysis of surface‐bound β‐GP was markedly suppressed on the (100) facet compared to the (002) facet (Figure 3B,D,I). This kinetic disparity is a direct consequence of the stronger adsorption on the (100) surface, where robust, multi‐point anchoring “locks” β‐GP into a rigid orientation. When coupled with the elevated surface packing density, this creates a congested interfacial microenvironment (also known as macromolecular crowding effects), which imposes significant steric hindrance and limits the accessibility of the scissile phosphate ester bond to the ALP active site. This observation aligns with the paradoxical inhibition of interfacial enzymatic activity under crowded conditions [29, 30, 31]. In contrast, the unstable adsorption on the (002) facet permits greater molecular mobility and facilitates enzymatic hydrolysis. Consequently, this facet‐selective enzymatic processing governs the spatiotemporal removal of the organic ligand, providing a potent mechanism to drive the hierarchical assembly observed in the EMS.

Interfacial energy acts as a fundamental driver in nanoparticle self‐assembly, particularly in OA [32, 33, 34]. For instance, anatase TiO_2_ nanoparticles preferentially align along high‐energy crystallographic planes (e.g., the {001} facets) to eliminate unstable interfaces and minimize total Gibbs free energy [35]. In our case, MD simulations revealed that the intrinsic interfacial energy anisotropy between the (002) and (100) facets of HAP (ratio = 1.5) is initially moderated by the facet‐selective adsorption of β‐GP (ratio = 1.3). Crucially, the differential hydrolysis kinetics subsequently amplify this interfacial energy contrast. The rapid, near‐complete hydrolysis on the (002) facet, juxtaposed with the persistent passivation on the (100) facet, elevates the interfacial energy ratio to a theoretical maximum of ∼2.1 (Figure 3K). These results were further corroborated by the interfacial energies calculated from water contact angle measurements for the (002) and (100) facets (Figure S15, Table S1, and Text S1). This enzyme‐driven amplification of energetic disparity serves as a dynamic gate for the sequential assembly process. To minimize Gibbs free energy, HAP nanorods initially undergo *c*‐axis alignment through OA, a process that preferentially eliminates the high‐energy {002} facets. This is facilitated by the localized, rapid removal of β‐GP, which restores the pristine surface energy of these planes and provides the maximal thermodynamic drive for assembly. Concurrently, the temporally‐extended passivation of the (100) facets delays their participation in assembly, with their interfacial energy increasing only gradually as β‐GP is eventually cleaved. This spatiotemporal energy gradient dictates a strict assembly hierarchy, longitudinal alignment followed by lateral aggregation (Figure 3L), ensuring the formation of the highly ordered architectures observed experimentally.

To validate the pivotal role of enzymatic activity, we conducted a control experiment in which levamisole, a potent ALP inhibitor [18], was introduced after the reaction had been ongoing for 2 h. At this stage, HAP nanorods had already formed and initiated early‐stage assembly (Figure 1F,G). Upon the addition of levamisole, the catalytic hydrolysis of surface‐bound β‐GP was effectively abolished, which halted the directed assembly process and resulted in the random aggregation of HAP nanorods into an ill‐defined structure (Figure S16). We posit that the persistence of the β‐GP coating maintains both the (100) and (002) facets in a low‐energy state, thereby neutralizing the thermodynamic driving force required for OA. Taken together, these results confirm that interfacial energy modulation, governed by enzymatic turnover, is the primary driver behind the hierarchical assembly of HAP. This mechanistic insight provides a versatile blueprint for employing enzymatic‐control strategies to engineer advanced biomimetic materials for enamel restoration.

### 2.4 Reconstruction of the Enamel Hierarchical Architecture

Based on the above findings regarding the enzyme‐mediated hierarchical assembly of CaP, we applied EMS to the in vitro remineralization of enamel. Simulating early caries, the enamel was demineralized with phosphoric acid, which dissolved the enamel crystals and resulted in a characteristic fish‐scale‐like microstructure with loosely packed crystals (Figure 4A,B) [36]. When the acid‐etched enamel (AE) is immersed in the EMS, the remineralization of enamel occurs. At the early stage (within 1 h), spherical CaP particles with a continuous structure formed, which evolved into spindle‐shaped structures on the enamel surface by 3 h. At this stage, individual nanorods (36.41 ± 7.26 nm in length, 12.6 ± 1.48 nm in width; marked by yellow arrows), the primary building blocks, became discernible within the spindle‐shaped assemblies (228.48 ± 13.34 nm in length, 72.58 ± 9.06 nm in width; Figure 4C,D). After 6 h of remineralization, the spindle‐shaped HAP assemblies had grown further, reaching lengths of 441.76 ± 56.34 nm and widths of 134.92 ± 18.54 nm (Figure 4E). This growth likely proceeded via the continuous integration of primary nanorods, which remained distinguishable (indicated by yellow arrows) within the larger superstructures. By 48 h, sustained assembly and coalescence of the nanorods/assemblies resulted in the formation of a densified and highly ordered mineral layer, replicating the original structure of natural enamel (Figure 4F–H). Notably, the assembly behavior of HAP nanorods on the enamel substrate mirrors the mechanism observed in solution, suggesting that spatiotemporal enzymatic control governs the mineralization process across both environments. The EMS simultaneously repaired both rods and inter‐rods despite their divergent orientations. This capacity for orientation‐independent reconstruction highlights that the assembly of HAP nanorods is nanoscale‐specific and controllable, thereby enabling high structural fidelity for the construction of enamel with complex architectures (Figure 4I). Furthermore, the thickness of the newly formed HAP mineral layer, measured to be approximately 2 µm by atomic force microscopy (AFM) and optical profilometry, was determined by quantifying the height difference between the unrepaired and repaired zones (Figure 4J,K; Figure S17).

**FIGURE 4:**
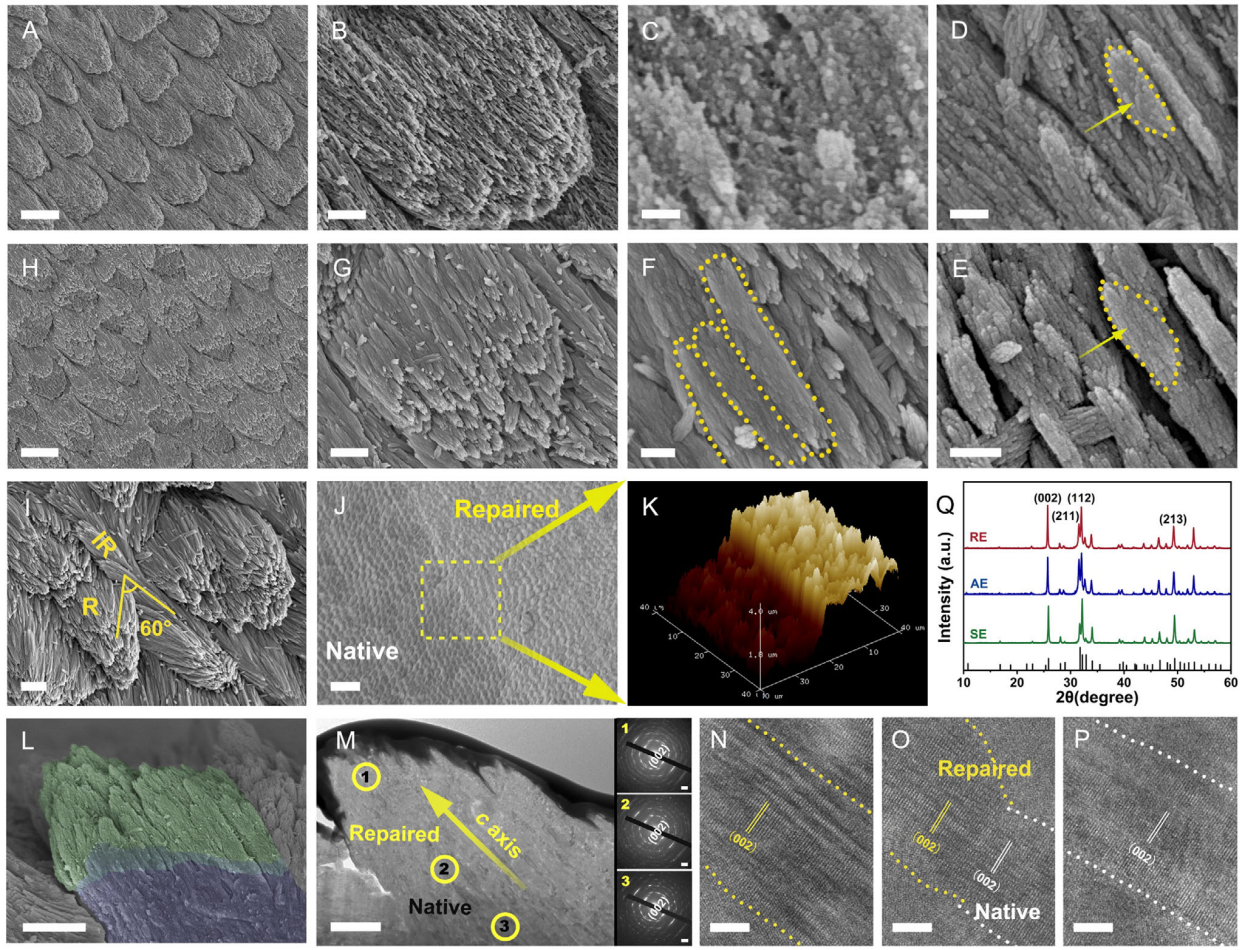
Biomimetic reconstruction of enamel hierarchical architecture via EMS. (A, B) SEM images of AE revealing a fish scale‐like structure and a high‐magnification view of enamel rods. Enamel remineralized in the EMS for 1 h (C), 3 h (D), 6 h (E), and 48 h (F‐H). (I) Simultaneous growth of rod (R) and interrod (IR) structures exhibiting diverse orientations. (J) SEM image juxtaposing acid‐etched and repaired enamel areas. (K) AFM image of enamel with one half repaired and the other half covered with nail polish as a control, used to confirm the thickness of the newly formed mineral layer. (L) Cross‐sectional view of RE after 48 h of remineralization, showing a well‐organized structure, with newly formed HAP adopting the native enamel rod orientation. The remineralized and native regions are highlighted in green and blue, respectively. (M) TEM image of a longitudinal section through the enamel, encompassing native and remineralized zones. The regions marked by yellow circles 1, 2, and 3 correspond to the remineralized, transitional, and native zones, respectively (transitional zone identified based on cross‐sectional SEM analysis of the same sample). SAED patterns corresponding to zones 1, 2, and 3 confirm that the long axis of the crystals is aligned with the crystallographic *c*‐axis of HAP. (N‐P) HRTEM images demonstrate continuous lattice fringes across all three zones. (Q) XRD analysis of SE, AE, and RE. Scale bars: 5 µm (A, H); 1 µm (B, G, I, L); 100 nm (C, D); 200 nm (E, F); 20 µm (J); 500 nm (M); 1 nm^−1^ (SAED patterns in (M)); 5 nm (N, O, P).

The interface between natural enamel and newly formed enamel‐like materials is observed via electron microscopy (EM), revealing seamless structural continuity at both the micro‐ and nanoscale. Cross‐sectional SEM imaging demonstrated that the newly formed HAP minerals exhibit pronounced alignment parallel to the longitudinal axis of the native enamel rods, substantiating the accurate replication of the organized structure of natural enamel (Figure 4L). To further resolve the interfacial architecture, an ultrathin section was extracted using focused ion beam (FIB) milling for TEM analysis. Longitudinal sections spanning the remineralized (Zone 1), transitional (Zone 2), and native (Zone 3) regions were systematically examined (Figure 4M). SAED patterns obtained from these three zones showed consistent (002) arc reflections, confirming that the *c*‐axis orientation of the HAP crystals is preserved. Moreover, the HRTEM images provided compelling evidence of a coherent transition from the native to the repaired zone. The presence of continuous lattice fringes and aligned (002) planes across the regions validates a unified crystallographic orientation and structural integration at the nanoscale (Figures 4N–P). While the newly formed HAP crystals exhibited a slightly larger diameter than their native counterparts (Figure 4O), this subtle morphological distinction serves as a reliable marker to delineate the repaired zone from the original substrate.

The phase composition and macro‐scale crystalline order of the repaired layer were characterized by X‐ray diffraction (XRD; Figure 4Q). The diffraction peaks observed at 25.9°, 31.7°, 32.1°, and 49.5° were assigned to the (002), (211), (112), and (213) crystallographic planes, respectively, which matched those of HAP crystals (Joint Committee on Powder Diffraction Standards (JCPDS) File No. 09–0432), confirming the formation of the HAP phase. To quantify the degree of crystalline alignment along the *c*‐axis, we calculated the intensity ratio of the (002) to (211) reflections (*I*_(002)_/*I*_(211)_) [4, 37]. The calculated ratios for sound enamel (SE), AE, and repaired enamel (RE) were 1.90, 1.06, and 1.74, respectively, which were all significantly higher than that for isotropic HAP powder (0.40; Based on JCPDS data). The proximity of the RE ratio to that of SE provides macro‐level evidence that the EMS‐induced mineral layer faithfully restores the crystallographic texture of natural enamel. Together, these results demonstrate a precise biomimetic replication spanning from the atomic lattice to the macroscopic mineral assembly.

The EMS technique restored enamel across diverse microstructural templates, including both honeycomb‐like and rod‐like architectures (Figure S18), underscoring the broad applicability of our approach in addressing a wide spectrum of enamel defects. Furthermore, to meet the requirements for practical clinical efficacy, we implemented a cyclic remineralization strategy. This approach facilitated the steady, incremental growth of the mineral layer at a rate of approximately 1 µm per day (Figure S19), demonstrating potential of the EMS for the customized treatment of early‐stage dental caries.

### 2.5 Evaluation of the Mechanical Properties

As the primary protective barrier of the tooth, the structural and mechanical integrity of enamel is indispensable for its biological function. We conducted a comprehensive evaluation of the enamel samples using a radar plot to integrate key mechanical and chemical descriptors, including hardness (*H*) and elastic modulus (*E*), coefficient of friction (*μ*), storage modulus (*E'*), damping coefficient (*tanδ*), and acid resistance (AR) (Figure 5A; Figure S20). The *H* and *E* were calculated from the load‐displacement curves collected via nanoindentation, using the established Oliver‐Pharr method [38]. The SE exhibited high *H* and *E* values (3.46 ± 0.25 GPa and 87.68 ± 5.37 GPa, respectively), consistent with previously reported values (Figure 5B) [39]. As expected, acid erosion substantially reduced the *H* and *E* of AE to 0.14 ± 0.04 GPa and 21.20 ± 4.06 GPa, respectively, primarily due to the loss of mineral density and the formation of a porous surface architecture [40]. Notably, after remineralization in the EMS, the RE achieved a near‐complete recovery of these properties (*H*: 3.25 ± 0.32 GPa; *E*: 86.67 ± 4.49 GPa), with no statistically significant deviation from SE (*p* > 0.05). Furthermore, the functional restoration of wear resistance was assessed via the coefficient of friction (*μ*) [41]. The *μ* of RE (0.16 ± 0.01) was mirrored that of SE (0.14 ± 0.01) and was significantly lower than that of AE (0.26 ± 0.03; Figure 5C). Meanwhile, under a load of 0.5 N, the repaired HAP layer exhibited almost no delamination, spallation, or detachment after microscratch testing, indicating strong interfacial bonding between the newly formed mineral layer and the underlying natural enamel (Figure S21). Additionally, the viscoelastic behavior of the enamel samples, a prerequisite for sustaining the cyclic stresses of mastication, was evaluated through dynamic mechanical analysis (DMA) [42] across a contact depth range of 100 to 350 nm. SE exhibited a storage modulus (*E'*) of 69.95 ± 2.36 GPa and a damping coefficient (*tanδ*) of 0.0647 ± 0.0059 (Figure 5D,E). Acid etching severely compromised the structural integrity of the enamel, resulting in a precipitous drop in *E'* to 10.55 ± 0.80 GPa and a sharp elevation of *tanδ* to 0.3754 ± 0.0101, reflecting a loss of elastic resilience. Remarkably, the EMS‐mediated remineralization facilitated a near‐complete restoration of these dynamic properties. The *E'* of the RE recovered to 67.96 ± 2.28 GPa (97% of the SE value), while the *tanδ* decreased to 0.0668 ± 0.0062, converging with the baseline performance of native enamel. These results indicate that the mechanical damping capacity and energy‐dissipation mechanisms of the enamel can be effectively restored to their functional physiological state.

**FIGURE 5:**
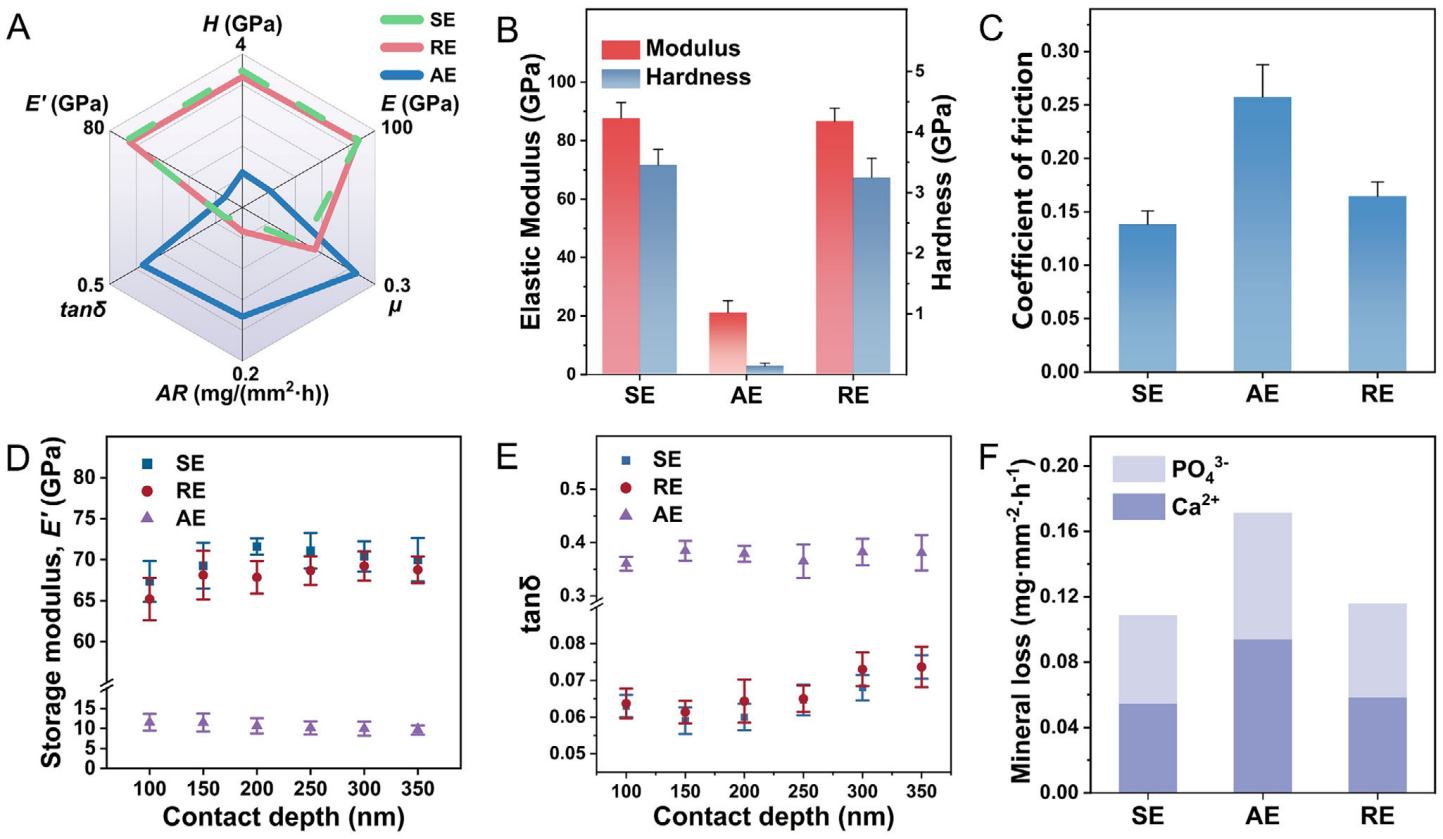
Mechanical performance and acid resistance of remineralized enamel. (A) Radar plot providing a holistic comparison of the mechanical and chemical properties of SE, AE, and RE. (B‐E) Quantitative analysis of mechanical parameters: (B) Hardness (*H*) and elastic modulus (*E*), (C) coefficient of friction (*μ*), (D) storage modulus (*E'*), (E) damping coefficient (*tanδ*). (F) Acid resistance (AR) of the various enamel samples, quantified by the mass of calcium and phosphate ions released per unit area per hour (mg·mm^−2^·h^−1^) under acidic challenge. AR values represent the dissolution kinetics, with lower rates indicating enhanced mineral stability.

AR is essential for maintaining the long‐term structural integrity and functional stability of enamel. To evaluate this, enamel specimens were subjected to an acidic challenge (pH 2.0) for 3 min, with the release kinetics of calcium (Ca^2+^) and phosphate (PO_4_^3−^) quantified via inductively coupled plasma‐mass spectrometry (ICP‐MS; Figure 5F). AE exhibited elevated ion release rates (Ca^2+^: 0.0941 ± 0.0067 mg·mm^−2^·h^−1^ and PO_4_^3−^: 0.0770 ± 0.0099 mg·mm^−2^·h^−1^), indicating high susceptibility to demineralization. In contrast, both SE and RE displayed significantly lower and comparable dissolution rates (For SE, Ca^2+^: 0.0548 ± 0.0027 mg·mm^−2^·h^−1^ and PO_4_^3−^: 0.0536 ± 0.0019 mg·mm^−2^·h^−1^; For RE, Ca^2+^: 0.0585 ± 0.0032 mg·mm^−2^·h^−1^ and PO_4_^3−^: 0.0571 ± 0.0043 mg·mm^−2^·h^−1^), suggesting that the EMS‐generated HAP layer effectively enhances the AR capacity of the demineralized enamel. Overall, these findings demonstrate that the EMS facilitates a near‐complete restoration of the mechanical and chemical properties of enamel, a feat enabled by the formation of densely packed HAP minerals and the reconstruction of the ordered hierarchical microstructure.

### 2.6 Enamel Restoration in Vivo

To evaluate the efficacy of the EMS strategy within the complex intraoral milieu, we encapsulated the EMS within a GelMA hydrogel matrix. This hydrogel provides a semi‐isolated microenvironment that maintains relatively stable remineralization conditions and sustains a localized pH of approximately 8.5 within the mineralization zone, which is favorable for ALP activity, while the surrounding oral environment remains at physiological pH. Meanwhile, the macroporous hydrogel provides interconnected aqueous pathways that facilitate the diffusion of CaP particles, including HAP nanorods, toward the enamel surface (Figure S22) [43, 44]. Preliminary CCK‐8 assays confirmed the excellent cytocompatibility of the EMS‐hydrogel (Figure S23). Before conducting in vivo experiments, we verified that the EMS‐hydrogel could remineralize enamel and reconstruct its hierarchical architecture in vitro by a hierarchical assembly of CaP particles (Figures S24 and S25). For preclinical validation, AE blocks were surgically fixed in the oral cavities of Sprague‐Dawley (SD) rats to evaluate the remineralization efficiency in vivo (Figure 6A). In this assay, ultrapure water (UPW) and the commercial fluoride varnish Duraphat served as the negative and positive controls, respectively. The AE treated with UPW showed no mineral deposition on the enamel surface (Figure 6B1–B3). Although Duraphat is widely used clinically for caries prevention, it only deposited spherical mineral aggregates on the enamel surface (Figure 6C1–C3). By contrast, EMS treatment resulted in the formation of a dense, highly organized HAP layer on the enamel surface that closely resembles natural enamel (Figure 6D1–D3). These findings were corroborated by optical profilometry, which revealed that the EMS facilitated the growth of a 1.73 µm thick mineral layer with high structural fidelity. No noticeable height change was observed in the UPW and Duraphat group (Figure 6E–G).

**FIGURE 6:**
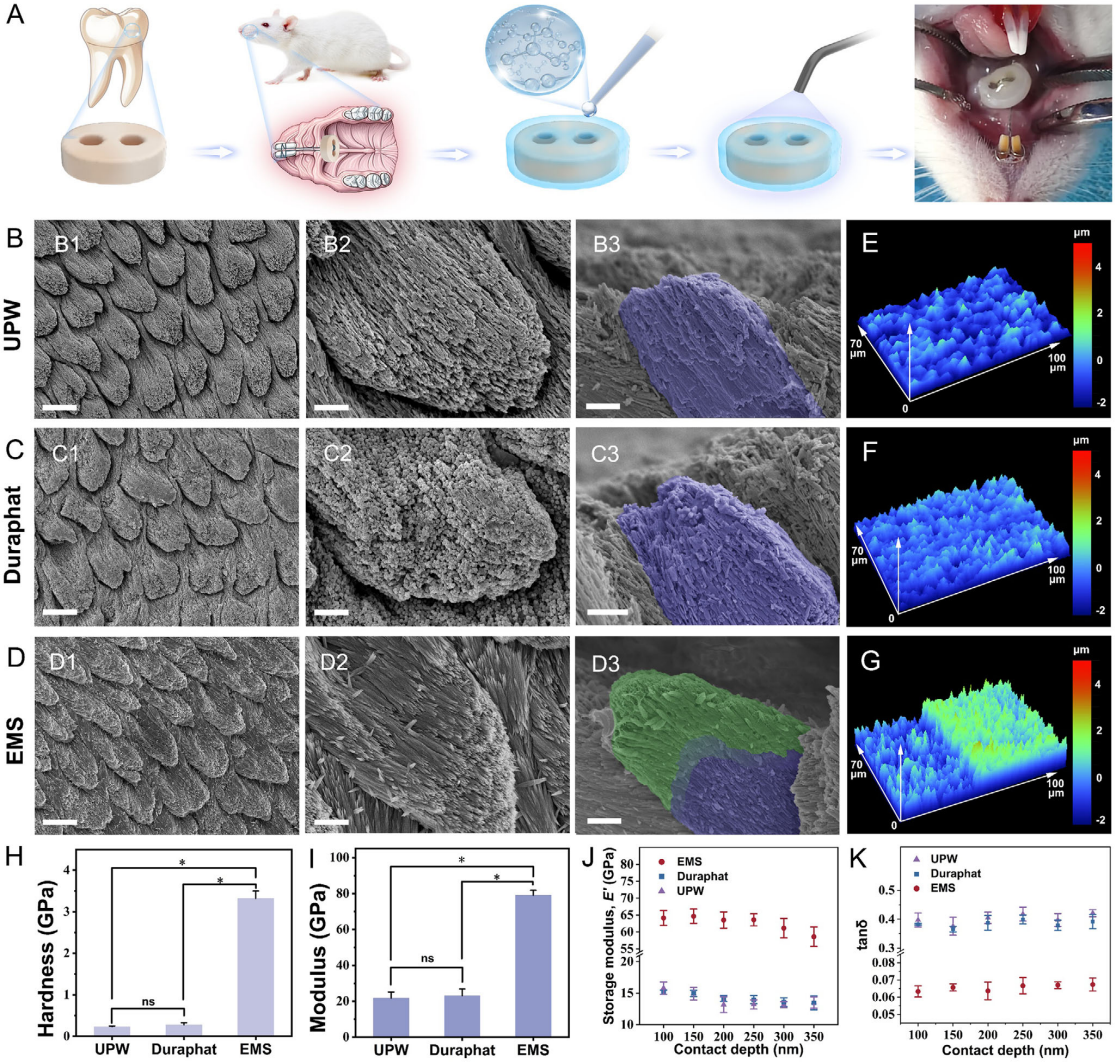
In vivo enamel repair via the EMS‐hydrogel. (A) Schematic workflow of the in vivo experimental protocol. AE blocks with two holes were intraorally fixed in SD rats. The EMS was applied to the enamel surface, light‐cured, and subsequently subjected to in vivo remineralization for 7 days. (B‐D) Representative SEM images of the enamel surface after 7 days of intraoral exposure following treatment with: (B) ultrapure water (negative control), (C) Duraphat (positive control), and (D) EMS‐treated enamel. Scale bars: B1, C1, D1: 5 µm; B2, C2, D2: 1 µm; B3, C3, D3: 500 nm. (E‐G) Optical profilometry analysis used to confirm the formation of the new mineral layer. One‐half of each specimen was repaired, while the other was masked with nail polish as a reference plane. (E‐G) correspond to the UPW, Duraphat, and EMS groups, respectively. (H‐K) Quantitative evaluation of enamel mechanical properties: (H) Hardness, (I) Elastic modulus, (J) Storage modulus, and (K) Damping coefficient. Data are presented as means ± standard deviations (N = 3). Statistical significance among groups was determined using one‐way analysis of variance (ANOVA) (*p < 0.05).

To further validate the clinical efficacy of the system, we systematically evaluated the mechanical properties of the in vivo remineralized layers (Figure 6H–K). The control treatments (UPW and Duraphat) failed to provide any significant mechanical reinforcement. The *H*, *E*, *E'*, and *tanδ* remained severely compromised for both the UPW group (*H* = 0.23 ± 0.02 GPa, *E* = 21.78 ± 3.33 GPa, *E' =* 13.96 ± 1.12 GPa, and *tanδ =* 0.40 ± 0.016) and the Duraphat group (*H* = 0.28 ± 0.05 GPa, *E* = 23.04 ± 3.78 GPa, *E' =* 14.20 ± 0.70 GPa, and *tanδ =* 0.38 ± 0.012). In striking contrast, the EMS‐hydrogel treatment can significantly enhance the mechanical strength of enamel, with values (*H =* 3.32 ± 0.17 GPa, *E =* 79.02 ± 2.82 GPa, *E' =* 62.59 ± 2.31 GPa, and *tanδ =* 0.066 ± 0.0017), reaching levels comparable to those of natural enamel. These results indicate that the EMS can circumvent the multifaceted challenges of the intraoral environment, such as salivary interference and mechanical abrasion, to achieve authentic, functional enamel regeneration in vivo.

## 3 Conclusion

Inspired by biomineralization, this study has developed an EMS platform for both in vitro and in vivo enamel remineralization, which can precisely reconstruct the complex structure of enamel and restore its mechanical strength to its natural level. In contrast to previous biomimetic strategies, which focused primarily on the nucleation and growth of CaP or the phase transformation of amorphous precursors, our EMS platform achieves enamel restoration via orchestrated hierarchical assembly of HAP. When the demineralized enamel was remineralized in the EMS, the enzymatic reaction triggers CaP particle formation and initiates the assembly process. By coupling the facet‐specific adsorption of β‐GP on HAP with its removal by ALP, the system generates a spatiotemporally dynamic interfacial energetic landscape across the HAP (100) and (002) facets. This landscape provides the essential thermodynamic driving force to steer the hierarchical assembly process, which is characterized by initial *c*‐axis‐oriented attachment followed by lateral aggregation, ultimately yielding micro‐scale bundles that replicate the organization structure of native enamel. This strategy represents a fundamental shift from simple mineral deposition toward the orchestrated, multi‐scale organization required for functional enamel restoration.

In the crystallization of biominerals, organic molecules often undergo facet‐specific adsorption, effectively passivating select crystal surfaces to facilitate oriented growth. For example, the hydrophilic C‐terminus of amelogenin preferentially binds to the HAP (100) facet, suppressing lateral expansion and thereby promoting longitudinal elongation along the *c*‐axis through a classical monomer‐by‐monomer addition pathway [26]. Unlike static facet‐specific passivation, the EMS introduces a dynamic regulatory mechanism. While β‐GP initially adsorbs onto and passivates both the (100) and (002) facets of HAP, its enzymatic hydrolysis is spatiotemporally differentiated across these planes, resulting in re‐exposure of high‐energy crystal surfaces such as the (002) facet and thereby generating a pronounced differential interfacial energy. Meanwhile, translational and rotational Brownian diffusion enables nanorod encounters and continuous exploration of mutual orientations [45, 46]. The progressive removal of surface‐adsorbed β‐GP reduces the interparticle repulsive barrier, allowing nanorods to approach sufficiently closely for attractive interactions to become effective. Among the accessible configurations, facet‐to‐facet arrangements that more effectively reduce the interfacial energy are preferentially stabilized once formed, thereby biasing the orientational distribution toward pre‐aligned states and facilitating subsequent OA. Together, the hydrolysis‐induced reduction in the colloidal interaction barrier and the resulting anisotropic interfacial‐energy landscape provide the driving force for pre‐alignment and oriented assembly of HAP nanorods along the c‐axis. Beyond enamel, the surface adsorption behavior of biomolecules onto HAP has been extensively studied in biomaterials contexts, where HAP surface microstructures significantly influence protein adsorption [47, 48, 49]. Furthermore, from a broader biomineralization perspective, this work offers fresh insights into the multifunctionality of enzymes associated with amelogenesis. Beyond their primary biochemical roles in matrix degradation or phosphate supply, this work suggests that these enzymes (e.g., MMP‐20, ALP) can actively shape the structure of the biomineral by modulating the interfacial energy. Such a mechanism may be ubiquitous in the formation of various hierarchical biostructures, where enzymatic activity acts as a temporal switch to regulate the assembly of mineral particles.

The OA pathway has long been recognized as a ubiquitous phenomenon in synthetic, biogenic, and geologic environments and extensively used to fabricate a diverse array of functional materials. During conventional OA‐mediated oriented growth, organic ligands typically bind firmly to particle surfaces to modulate interfacial energy. However, even after the assembly is complete, these ligands often remain entrapped within the resulting materials, potentially compromising the purity and structural integrity of the final mineral [50, 51]. In this study, the EMS‐mediated OA process initially utilizes β‐GP as a transient ligand to modulate the interfacial energy and stabilize the nanorod building blocks. Crucially, this organic modulator is subsequently degraded via in situ enzymatic hydrolysis, eliminating organic barriers and facilitating the seamless fusion of nanorods into a structurally integrated architecture. From this perspective, the EMS platform offers a versatile strategy for the fabrication of hierarchically structured materials with exceptional mechanical performance.

Following remineralization within the EMS, the newly formed HAP mineral layer exhibited structural and mechanical properties that were indistinguishable from those of natural enamel. The regenerated minerals maintained seamless crystallographic continuity across the native‐repaired interface. However, the remineralization efficiency of this EMS‐based strategy is currently limited by the intrinsic kinetics of crystal nucleation and growth. Consequently, a single treatment cycle yields a repaired layer only several micrometers thick, and although repeated treatments can increase the repair thickness, the overall efficiency remains limited. One of future studies should therefore focus on accelerating crystallization kinetics to enable the rapid and robust restoration of clinically relevant, large‐scale enamel lesions. For clinical translation, additional challenges need also be addressed, including the effective removal of biofilms from the tooth surface, potential effect of the salivary components (e.g., proteins, mucins), and the influence of mechanical stresses in the oral environment. Additionally, longer‐term studies with larger cohorts are warranted to evaluate treatment durability and safety. Systematic assessment of these factors will be essential for further optimizing the EMS‐hydrogel treatment strategy and ensuring reliable and sustained enamel repair in future clinical applications. If these issues are adequately addressed, the EMS‐hydrogel system may have potential for clinical application as a convenient chairside treatment, with an estimated application time of approximately 5–10 min and a projected raw material cost of less than US$2 per single‐tooth treatment.

## 4 Materials and Methods

### 4.1 Materials

All reagents were used directly without additional purification, unless otherwise specified. Calcium chloride dihydrate (CaCl_2_·2H_2_O, 99.0%), β‐glycerophosphate disodium salt pentahydrate (β‐GP), 4‐(2‐hydroxyethyl)piperazine‐1‐ethanesulfonic acid (HEPES, 99%), ammonium vanadate (NH_4_VO_3_, 99%), and dipotassium hydrogen phosphate (K_2_HPO_4_, 99%) were purchased from Sigma‐Aldrich. Alkaline phosphatase (ALP; bovine intestinal mucosa, ≥ 10 units/mg solid) was purchased from Nantong Feiyu Biological Technology. Tris(hydroxymethyl)aminomethane (Tris base, 99.5%), sodium chloride (NaCl, 99.5%), (3‐aminopropyl)triethoxysilane (APTES, 99%), acetone (99.5%), calcein, 10‐methacryloyloxydecyl dihydrogen phosphate (MDP, 98%), para‐nitrophenyl phosphate (pNPP, 98%), alkaline phosphatase buffer, and ALP stabilizing buffer were purchased from Aladdin. Sodium fluoride (NaF, 99%), phosphoric acid (H_3_PO_4_, 85%), nitric acid (HNO_3,_ 65%), sulfuric acid (H_2_SO_4_, 95%), and ethanol (99.7%) were purchased from Sinopharm Chemical Reagent. Deuterium oxide (D2O, 99.8 atom% D) was purchased from J&K. Levamisole hydrochloride (C_11_H_12_N_2_S·HCl, >99.0%) was purchased from TCI. Gelatin Methacrylate (GelMA‐60) and lithium phenyl‐2,4,6‐trimethylbenzoylphosphinate photoinitiator (LAP) were purchased from EFL. Ammonium molybdate tetrahydrate ((NH_4_)_6_Mo_7_O_24_, 99.8%), and para‐nitrophenol (pNP, 99%) were purchased from Macklin. Uranyl acetate dihydrate was purchased from Beijing Xinxing Bairui Technology Co., Ltd. Cy3‐labeled β‐GP (Cy3‐β‐GP) and Cy5‐conjugated alkaline phosphatase (Cy5‐ALP; 95%) were purchased from Xian Ruixi Biological Technology. These compounds were used as fluorescent probes in this study. All solutions were prepared using ultrapure water (UPW, 18.2 MΩ·cm) and filtered through 0.22 µm polyethersulfone membranes (Millipore) before use. Healthy human molars were obtained from Stomatology Hospital, School of Stomatology, Zhejiang University School of Medicine. The collection procedure was conducted with informed consent of the patients and was approved by the institutional ethics committee (Approval No. 2024–095(R)). The enamel slices were sectioned using a water‐cooled diamond saw, mechanically polished through a sequential series of silicon carbide papers (600, 1200, and 2400 grit), and subsequently ultrasonicated in UPW for 5 min.

### 4.2 Observation of Mineral Evolution Within the EMS

The morphological evolution of CaP particles within the EMS was monitored by using TEM. The EMS contained 20 mM CaCl_2_, 2 µg/mL ALP, 3 mM β‐GP, 150 mM NaCl, 0.77 mM NaF, and 10 mM Tris buffer at pH 8.5. At a designated time, 5 µL of the reaction solution was dropped onto a TEM grid, gently rinsed with ethanol, and subsequently air‐dried at room temperature (RT) before imaging. Throughout this study, the ALP concentration was fixed at 2 µg/mL. At lower concentrations (e.g., 1 µg/mL), phosphate generation was insufficient to support efficient mineralization within the experimental timeframe. In contrast, a higher ALP concentration (e.g., 5 µg/mL) accelerated the mineralization process through rapid phosphate generation, resulting in the formation of less ordered HAP structures (data not shown).

### 4.3 In Situ Real‐Time Confocal Laser Scanning Microscopy (CLSM)

To visualize the enzymatic hydrolysis of Cy3‐β‐GP by Cy5‐ALP on HAP crystals, real‐time fluorescence imaging was performed using a CLSM (LSM 980, Carl Zeiss AG, Germany). The HAP nanocrystals collected from EMS after 24 h were transferred to the central channel of an ibidi μ‐Slide I^0.4^ Luer 3D (Cat. No. 87176, Germany), and then prestained with 10 µM calcein in DMSO for 20 min at RT. After staining, the samples were gently washed three times with DMSO. Initially, 50 µL of 10 µM Cy3‐β‐GP solution was added to the left reservoir of the μ‐Slide. After 5 min of incubation to allow for substrate adsorption, fluorescence imaging was conducted. Subsequently, 50 µL of 10 µM Cy5‐ALP was added to the right reservoir, and after a further 5 min, fluorescence images were acquired. In addition, the enzymatic hydrolysis of Cy3‐β‐GP adsorbed onto the (100) and (002) facets of bulk HAP crystals was visualized using CLSM (LSM 980, Carl Zeiss AG, Germany). Millimeter‐sized HAP crystals extracted from a natural mineral source (Murmansk Oblast, Russia) were sectioned using a water‐cooled diamond saw to expose defined crystallographic planes. Following ultrasonication in UPW for 3 min, the sections were placed in confocal imaging dishes. The samples were first incubated with 200 µL of 10 µM Cy3‐β‐GP for 5 min, followed by fluorescence imaging. Subsequently, 200 µL of 1 mg/mL ALP solution was added, followed by another 5 min incubation and a second round of imaging. Fluorescence intensity was quantified using ZEN software (version 3.8.99, Carl Zeiss AG, Germany).

### 4.4 Confocal Raman Microscopy

To detect the interaction between β‐GP and CaP minerals, Raman spectroscopy measurements were performed using a Horiba LabRAM HR Evolution system (Japan) with a 532 nm excitation laser. The samples were collected from EMS at a designated time. To isolate the mineral, the reaction medium was centrifuged at 12,000 rpm for 10 min (Centrifuge 5810R, Eppendorf, Germany) and washed three times with anhydrous ethanol. Subsequently, the resulting precipitates were dried under vacuum at 70°C for 24 h. Raman spectra were acquired over a spectral range of 400–4000 cm^−1^. Spectral reproducibility was confirmed by collecting measurements at three distinct points on each sample.

### 4.5 Fluorescence Cross‐Correlation Spectroscopy (FCCS) Experiments

After 24 h of incubation, HAP crystals were collected from EMS and immobilized in a glass‐bottom imaging dish (Cat. No. D35‐20‐1.5H, Cellvis, USA). The HAP were prestained with calcein (5 µM) for 20 min at RT and then gently washed three times with UPW to remove any unbound fluorophores. Subsequently, Cy3‐β‐GP was introduced at a final concentration of 5 nM. FCCS measurements were performed at RT on an Olympus FV4000 CLSM (Japan) equipped with a CX‐D20 FCCS module (Zolix, China). Calcein and Cy3 were simultaneously excited with 488 nm and 550 nm lasers, respectively. After establishing the baseline cross‐correlation, ALP was added (1 mg/mL final concentration) and allowed to react for 5 min, after which a second set of FCCS data was acquired under identical instrument settings. The acquired cross‐correlation (CC) curves were fitted to a model for a single diffusing species (Equation 1):

(1) $$G\;\left(\tau \right)=G\left(0\right)\frac{1}{1+\frac{\tau }{\tau_{D}}}\sqrt{\frac{1}{1+\frac{\tau }{s^{2}\tau_{D}}}}$$

where *G(0)* represents the cross‐correlation amplitude at zero lag time, *τ_D_* is the correlation time, and *s* is the structure factor. The structure factor was fixed at ∼5.0, determined through systematic calibration using a Cy3 reference solution with a known diffusion coefficient. Data analysis and curve fitting were performed using the built‐in software suite of the CX‐D20 module.

### 4.6 Details of MD Simulations

All the MD simulations were performed using the GROMACS 4.6.7 package in the NVT ensemble. The visualization was generated via the VMD package. Periodic boundary conditions were applied in all three dimensions. The CHARMM27 all‐atom force field was used to describe the β‐GP molecules, while the force field parameters for HAP were adopted from the report of Hauptmann et al. [52]. A time step of 2 fs was used, and the system temperature was maintained at 310 K using the Nosé‐Hoover thermostat. Long‐range electrostatic interactions were calculated via the particle mesh Ewald (PME) summation, with a cutoff of 1.3 nm for the separation of the direct and reciprocal space summation. The cutoff distance for the van der Waals interaction was 1.3 nm. The parameters of the Lennard‐Jones potential for the cross interactions between non‐bonded atoms were obtained from the venerable Lorentz‐Berthelot combination rule.

Each simulation system contained ∼10500 atoms within a box of dimensions about 3.7 × 9.3 × 3.4 nm^3^. The model consisted of a HAP crystal (around 3.7 × 3.3 × 3.4 nm^3^), ∼2200 TIP3P water molecules, and 20 β‐GP molecules. Na^+^ was added to each system to ensure electrical neutrality. After the adsorption of β‐GP on the HAP substrate, a 50 ns simulation was performed for the interfacial energy calculation. The initial 40 ns was discarded as equilibration in each simulation trajectory, and the subsequent 10 ns was utilized as the production run for analysis. To ensure the statistical significance of the obtained results, five parallel MD trajectories were carried out for all the simulation systems. The adsorption energy of the β‐GP molecule on HAP (100)/(002) face was calculated according to Equation (2):

(2) $$E_{adsorption,\beta -GP}=E_{HAP+\beta -GP\;}-\left(E_{HAP\;}+E_{\beta -GP}\right)$$

The interfacial energy *γ* between HAP and water (including HAP‐water and HAP + β‐GP‐water systems) was calculated according to Equation (3), following established protocols [53]:

(3) $$\gamma_{HAP-water}=\frac{E_{HAP+water}-\left(E_{bulk,\;HAP\;}+E_{bulk,\;water}\right)}{2A}$$

where *A* is the interfacial area between HAP and the water solution. All the radial distribution function (RDF) profiles were calculated using the radial distribution function *g(r)*, implemented in VMD, and the corresponding simulation snapshots were also visualized using VMD.

### 4.7 Enamel Remineralization

To simulate early‐stage caries lesions, enamel slices were acid‐etched with 37% (w/w) phosphoric acid for 30 s to create a demineralized surface. The specimens were subsequently cleaned via ultrasonication for 5 min to remove surface debris. A control zone was established on each specimen by covering half of the demineralized enamel surface with acid‐resistant nail varnish. Remineralization was conducted by immersing the enamel samples in the EMS at 37°C for a designated time. Following the treatment, the samples underwent ultrasonication in either absolute ethanol or UPW for 5 min to ensure the removal of any loosely adherent or non‐specifically bound mineral precipitates, leaving only the newly formed mineral layer for subsequent characterization.

### 4.8 Preparation of an Ultrathin Enamel Sample

For TEM observation of the ultrastructure of remineralized enamel, ultrathin sections were prepared using a dual‐beam focused ion beam‐scanning electron microscope (FIB‐SEM; Quanta 3D FEG, FEI, USA) equipped with a liquid gallium ion source. Regions of interest were first identified by preliminary SEM imaging. To prevent ion‐beam‐induced damage and preserve the surface morphology, a protective platinum (Pt) layer was locally deposited onto the target site before milling. A cross‐sectional lamella was subsequently excavated, thinned to an electron‐transparent thickness of approximately 100 nm, and extracted using an in situ micro‐manipulator. Finally, the lamella was mounted onto a copper TEM grid for HRTEM and SAED analysis.

### 4.9 Scanning Electron Microscopy (SEM)

Surface morphology of the samples was observed using a SEM (SU8010, Hitachi, Japan). Before imaging, all specimens were sputter‐coated with a thin gold/palladium (Au/Pd) conductive layer for 90 s using an ion sputtering coater (MC1000, Hitachi, Japan) to improve surface conductivity. SEM imaging was performed at an accelerating voltage of 4 kV in secondary electron imaging mode.

### 4.10 Atomic Force Microscopy (AFM)

To precisely determine the thickness of the newly formed HAP mineral layer on the enamel surface, the enamel specimen was prepared with half of the surface left unremineralized as a reference plane, and the other half remineralized in the EMS. The specimens were examined using AFM (Dimension Icon, Bruker, Germany) in PeakForce Tapping mode with a silicon cantilever probe (RTESPA‐300‐10, Bruker; nominal resonance frequency: 300 kHz, spring constant: 40 N/m). AFM images were acquired at a scan rate of 1.0 Hz with a resolution of 256 × 256 pixels over multiple 40 × 40 µm^2^ regions. The acquired data were subsequently processed with NanoScope Analysis v2.0.

### 4.11 Transmission Electron Microscopy (TEM)

TEM and HRTEM were performed using either a JEOL JEM‐F200 microscope equipped with dual Super‐EDS detectors or a Hitachi HT 7700 (Japan). All images were acquired at an accelerating voltage of 80 kV.

### 4.12 X‐ray Diffraction (XRD)

The XRD spectra of the samples were acquired using a Rigaku Ultima IV diffractometer (Rigaku Corporation, Japan) with Cu Kα radiation (λ = 1.542 Å). Measurements were performed in continuous scan mode at 40 kV and 40 mA over a 2θ range of 10°‐70°, with a step size of 0.02°.

### 4.13 Nanoindentation

Nanoindentation tests were conducted on SE (N = 3), AE (N = 3), and RE (N = 3) enamel samples using an iNano instrument (KLA, USA) equipped with a Berkovich diamond tip (tip radius ∼20 nm). The indenter was calibrated against a fused silica standard before testing. Hardness and elastic modulus were determined by the continuous stiffness measurement method under the following conditions: target load, 50.0 mN; surface approach velocity, 100.0 nm/s; strain rate, 0.200 s^−1^; and Poisson's ratio, 0.28. On each sample, 30 indentations were performed, and the hardness and elastic modulus were calculated from the load‐displacement curves.

### 4.14 Dynamic Mechanical Analysis (DMA) Test

The DMA of enamel samples was performed on an ultra‐high‐resolution nanoindenter (UNHT^3^, Anton Paar, Austria) equipped with a Berkovich diamond indenter (tip diameter ∼20 nm) to obtain storage modulus (*E'*) and damping coefficient (*tanδ*). Testing was carried out under displacement control at a frequency of 15 Hz. Samples were subjected to oscillatory loading starting from an initial dynamic force amplitude of 20 µN, with the force amplitude linearly ramped from 20 µN to a maximum force of 10 mN. Data were collected from at least three independent tests.

### 4.15 Micro‐scratch Tests

Micro‐scratch tests were performed on enamel samples using a micro‐tribometer (UMT‐5, Bruker, USA). Reciprocating sliding tests were performed using 3 mm diameter GCr15 steel counter‐balls (high‐carbon chromium bearing steel), with a normal load of 15 N and a reciprocating frequency of 1.25 Hz for 30 min. The coefficient of friction was recorded in real time.

### 4.16 Acid Resistance (AR) Tests

Enamel samples with a 2 × 2 mm^2^ window were immersed in 15 mL of 50 mM citric acid buffer (pH 2.0) for 3 min. The concentrations of calcium and phosphate ions released into the acid solution were quantified using inductively coupled plasma‐mass spectrometry (ICP‐MS; NexION 300X, PerkinElmer, USA). The AR was evaluated based on the release rate of total minerals, calculated as the sum of released calcium and phosphate ions, and expressed in mg/(mm^2^·h). Each experimental group contained at least three replicates (N ≥ 3).

### 4.17 Enamel Repair in the EMS‐Hydrogel

First, the hydrogel prepolymer solution was prepared by dissolving 10 w/v% GelMA in 10 mM Tris buffer (pH 8.5) under gentle stirring at 60°C for 10 min. Subsequently, the following components were added into the hydrogel prepolymer solution: 1 mM MDP, 20 mM CaCl_2_, 10 mM β‐GP, 0.77 mM NaF, 4 µg/mL ALP, 0.25 w/v% LAP. The resulting mixture was dropped onto enamel surfaces, photo‐crosslinked under UV light (365 nm, 5 mW/cm^2^) for 60 s, and subsequently incubated at 37°C for 2 days in artificial saliva (AS) containing 1.67 mM CaCl_2_, 1 mM Na_2_HPO_4_, 150 mM NaCl, 10 mM HEPES (pH 7.0). For fluoride ion‐containing AS group, acid‐etched enamel samples were directly immersed in AS supplemented with 0.77 mM NaF under the same incubation conditions, without prior EMS‐hydrogel treatment.

### 4.18 Enamel Repair in Vivo

Male SD rats (4 weeks, 100–120 g, N = 3 per group, RRID: RGD_734476) were used for in vivo enamel repair, approved by the Animal Experimental Ethics Committee of Zhejiang University (ZJU20250220). Enamel blocks (3 × 2 × 1 mm^3^), acid‐etched with 37% H_3_PO_4_ for 30 s, had two holes drilled for fixation. A 0.2 mm stainless steel wire threaded through these holes secured the blocks to the upper central incisors of the SD rats. Different remineralizing agents (100 µL) were applied to the etched enamel surfaces: negative controls treated with UPW, positive controls were treated with Duraphat (Colgate, USA), and experimental specimens were treated with EMS‐hydrogel, which was subsequently photo‐crosslinked under UV light (365 nm, 5 mW/cm^2^) for 60 s. Enamel surfaces were fully exposed to native saliva in the oral cavity, enabling human‐like remineralization. Rats were fed soft food to prevent surface damage. After 7 days, samples were removed, sonicated in deionized water, and analyzed using SEM and nanoindentation.

### 4.19 Statistical Analysis

One‐way ANOVA was used for statistical analysis using OriginPro 2023b software (OriginLab, Northampton, MA, USA). Statistical significance was indicated as ^∗^ *p* < 0.05. Error bars represent the mean ± SD, as indicated in the figure caption.

## Author contributions

Z.C., C.Y.S., and R.K.T initiated this project. C.Y.S., Z.H.T., Z.S.Z., Y.Y.Z., W.Z.W., C.R.Z., C.L.,T.S., and Q.Y.L. designed and performed the experiments. W.J.J., Z.M.L., W.F.F., Y.H. and Z.J.X. supervised the experiment design and data interpretation. C.Y.S., R.K.T. and Z.C. supervised and supported the project. Z.H.T., Z.S.Z., C.Y.S., R.K.T. and Z.C. wrote the manuscript. All authors reviewed and approved the manuscript.

## Conflicts of Interest

The authors declare no conflicts of interest.

## Supporting information




**Supporting File**: advs78180‐sup‐0001‐SuppMat.docx.

## Data Availability

The data that support the findings of this study are available from the corresponding author upon reasonable request.
