# Supplementary material for: Spatiotemporal Regulation of Hierarchical Assembly of Hydroxyapatite for Enamel Remineralization
Source: Adv Sci (Weinh). 2026 Oct 8:e78180. Online ahead of print. doi: 10.1002/advs.78180 (PMC13650029; doi:10.1002/advs.78180)
Supplement: Supplementary file 1 [file ADVS-9999-e78180-s001.docx]

Supplementary Materials for

**Spatiotemporal Regulation of Hierarchical Assembly of Hydroxyapatite for Enamel Remineralization**

*Zhenhang Tang^1#^, Zhisen Zhang^2#^, Yuanyu Zhou^1^, Wenzhi Wu^1^, Congrui Zhao^1^, Chang Liu^1^, Tong Shi^1^, Wenjing Jin^1^, Zhaoming Liu^3^, Weifeng Fang^3^, Qingyun Lin^4^, Yi He^5^, Zhijian Xie^1^, Ruikang Tang^3^*, Changyu Shao^1^*, Zhuo Chen^1^**

^1^Stomatology Hospital, School of Stomatology, Zhejiang University School of Medicine, Zhejiang Provincial Clinical Research Center for Oral Diseases, Zhejiang Key Laboratory of Oral Biomedical, Engineering Research Center of Oral Biomaterials and Devices of Zhejiang Province, Hangzhou 310016, China.

^2^Department of Physics, Research Institute for Biomimetics and Soft Matter, Jiujiang Research Institute and Fujian Provincial Key Laboratory for Soft Functional Materials Research, Xiamen University, Xiamen 361005, China.

^3^Department of Chemistry, Zhejiang University, Hangzhou 310058, China.

^4^Center of Electron Microscopy, State Key Laboratory of Silicon and Advanced Semiconductor Materials, School of Materials Science and Engineering, Zhejiang University, Hangzhou, 310027, China.

^5^College of Chemical and Biological Engineering, Zhejiang University, Hangzhou 310058, China.

#These authors contributed equally to this work.

* Correspondence to: rtang@zju.edu.cn; shaocy@zju.edu.cn; zoechen@zju.edu.cn

**Table of Contents.**

Supplementary Method

Supplementary Figures S1 to S25

| Figure | Title | Related to |
| --- | --- | --- |
| Figure S1 | Spatiotemporal distribution of alkaline phosphatase (ALP) in developing teeth | Section 2.1 |
| Figure S2 | Characterization of ALP-mediated β-GP hydrolysis and mineralization initiation | Section 2.1 |
| Figure S3 | Interfacial association between CaP clusters and β-GP characterized by AFM-IR | Section 2.1 |
| Figure S4 | Raman spectroscopic analysis of the EMS mineralization process | Section 2.1 |
| Figure S5 | Evolution of CaP mineral in the absence of ALP | Section 2.1 |
| Figure S6 | Elemental mapping of the evolving CaP particles | Section 2.1 |
| Figure S7 | Real-time HS-AFM visualization of the in situ self-assembly of CaP particles into spindle-shaped architectures within the EMS | Section 2.1 |
| Figure S8 | Ultrastructural characterization of natural enamel rods | Section 2.1 |
| Figure S9 | Morphological evolution and phase transformation of CaP minerals in AS | Section 2.1 |
| Figure S10 | TEM characterization of mineral formation in EMS without F- | Section 2.1 |
| Figure S11 | Solid-state 13C NMR analysis of the CaP minerals | Section 2.1 |
| Figure S12 | Synthesis, characterization, and functional validation of fluorophore -conjugated ALP and β-GP | Section 2.2 |
| Figure S13 | Morphological and crystallographic characterization of millimeter -sized HAP crystals | Section 2.3 |
| Figure S14 | Radial distribution function (RDF) analysis of β-GP on the HAP surfaces | Section 2.3 |
| Figure S15 | WCA measurements on HAP crystal facets | Section 2.3 |
| Figure S16 | Disruption of hierarchical assembly of HAP nanorods via inhibition of β-GP hydrolysis | Section 2.3 |
| Figure S17 | Optical profilometry analysis of enamel remineralization within the EMS | Section 2.4 |
| Figure S18 | Restoration of enamel across distinct microstructural orientations | Section 2.4 |
| Figure S19 | Escalation of remineralization thickness via a cyclic treatment strategy | Section 2.4 |
| Figure S20 | Comprehensive mechanical characterization of enamel samples | Section 2.5 |
| Figure S21 | SEM images of the scratch track on EMS-remineralized enamel | Section 2.5 |
| Figure S22 | Microstructure of the GelMA hydrogel used for EMS loading | Section 2.6 |
| Figure S23 | Cytocompatibility assessment of the EMS-hydrogel | Section 2.6 |
| Figure S24 | SEM characterization of CaP mineral structure within the EMS hydrogel | Section 2.6 |
| Figure S25 | Enamel remineralization facilitated by the EMS hydrogel | Section 2.6 |

Supplementary Table

Supplementary Text

Supplementary Reference

Supplementary Method

**1.1 Immunofluorescence Analysis of ALP in Developing Incisors**

Mandibular incisors from adult mice were harvested and fixed in freshly prepared 4% paraformaldehyde (PFA) at 4^o^C for 24 h. and decalcified in EDTA. The samples were then decalcified in 10% EDTA (pH 7.4) at 4^o^C for 3-4 weeks, with the decalcification solution changed every two days. After dehydration through a graded ethanol series, the tissues were embedded in paraffin. 5-μm-thick longitudinal sections were prepared. For immunofluorescence, sections were deparaffinized in xylene and rehydrated. Antigen retrieval was performed in 10 mM citrate buffer (pH 6.0) at 95^o^C for 15 min. To block non-specific binding, sections were incubated with 5% normal goat serum in PBS for 1 h at room temperature (RT). The sections were then incubated overnight at 4^o^C with a primary antibody against Alkaline Phosphatase (ALP; Servicebio, GB113915, RRID: AB_3754630). After three washes with PBS, sections were incubated with an Alexa Fluor 594-labeled Goat Anti-Rabbit IgG (Servicebio, GB28301, RRID: AB_3739944) in the dark for 1 h at RT. Nuclei were counterstained with DAPI (Beyotime, C1006) for 5 min. Finally, the sections were mounted with anti-fade mounting medium and visualized using a fluorescence microscope (CLSM; Nikon A1, Tokyo, Japan). To verify staining specificity, negative controls were processed in parallel by omitting the primary antibody.

**1.2 Immunofluorescence Staining of Mature Molars for ALP**

Mature molars were harvested and fixed in pre-cooled 70% ethanol at -20^o^C overnight. To preserve the mineralized structure, the teeth were sectioned into 500-μm-thick slices using a precision diamond saw. The resulting slices were sequentially dehydrated and fixed in 70% ethanol and acetone at -20^o^C for 25 min each. For immunostaining, slices were washed with PBS containing 0.1% Tween-20 (PBST) and blocked with 5% normal goat serum in PBST (0.05% Tween-20) for 30 min at RT. The slices were then incubated with an alkaline phosphatase recombinant rabbit monoclonal antibody (HUABIO, ET1601, RRID: AB_3069604) for 90 min at RT. After thorough washing, slices were incubated with Alexa Fluor 488-labeled Goat Anti-Rabbit IgG (Abcam, ab150077, RRID: AB_2630356) for 1 h at RT in the dark. Finally, the slices were washed and mounted with Fluoromount-G™ (Yeasen, 36307ES08). Unstained sections served as controls to define the baseline autofluorescence, allowing for precise adjustment of the imaging threshold. Images were captured using a KEYENCE BZ-X800 (Japan) fluorescence microscope.

**1.3 Nuclear Magnetic Resonance (NMR)**

Samples were prepared by dissolving the target compound in deuterium oxide (D_2_O) to obtain a concentration of 10 mM. Liquid-state NMR experiments were performed on a Bruker Avance III HD 600 MHz spectrometer (Bruker BioSpin, Germany). The data were processed using the MestReNova software (version 14.0, Mestrelab Research, Spain).

**1.4 Phosphate Measurement**

Samples were analyzed by vanadate-molybdate spectrophotometry at 430 nm. A standard curve (0-50 μg/mL PO_4_^3-^) was prepared by mixing PO_4_^3-^ stock (100 μg/mL in 0.5% H_2_SO_4_) with 2.0 mL reagent (NH_4_VO_3_, (NH_4_)_6_Mo_7_O_24_, and HNO_3_), diluting to 10 mL. A 5 mL of mineralized solution collected from EMS was acidified with 200 μL H_2_SO_4_, treated with 2.0 mL reagent, and diluted to 10 mL. Absorbance was read after 10 min by using a chromometer (AQ3700, Thermo Fisher Scientific, USA) with a measuring range of 0.05 to 80 ppm.

**1.5 Atomic Force Microscopy-Infrared Spectroscopy (AFM-IR)**

Freshly cleaved mica was coated with a thin Au/Pd layer to minimize substrate interference during AFM-IR measurements. Subsequently, 100 μL of the solution was dropped onto the mica substrate, followed by sequential washing with UPW three times and ethanol three times. AFM-IR was performed on a nanoIR2-FS system (Anasys Instruments, USA). AFM images were acquired in contact mode at a scan rate of 0.8 Hz with gold-coated probes (PR-EX-nIR2-10; Anasys Instruments) having a spring constant of 0.07-0.4 N/m and a resonant frequency of 13 ± 4 kHz. Nanoscale IR spectra were acquired directly across the range of 1961-909 cm^-1^ with a resolution of 2 cm^-1^/point and 128 coverages. IR spectral maps were generated by monitoring the peak-to-peak deflection at specific wavenumbers, using 16 coverages, 256 pt. resolution for X and Y.

**1.6 Fourier Transform Infrared Spectroscopy (FTIR)**

FTIR spectra of the samples were collected using a Nicolet iS20 spectrometer (Thermo Fisher Scientific, USA). Measurements were performed over the range of 400 to 4000 cm^-1^ with a resolution of 4 cm^-1^ and 32 scans per spectrum.

**1.7 Mineralization System in the Absence of ALP**

Control groups were established using a chemical formulation identical to the EMS but with the purposeful exclusion of the ALP enzyme. To maintain phosphate availability in the absence of enzymatic cleavage, inorganic phosphate was incorporated into the medium. The reaction medium was composed of 20 mM CaCl_2_, 0.2 mM K_2_HPO_4_, 2.8 mM β-GP, 150 mM NaCl, 0.77 mM NaF, and 10 mM Tris buffer at pH 8.5. To track the evolution of the mineral phase, aliquots were sampled at designated times. For TEM characterization (HT-7820, Hitachi, Japan), a 5 μL droplet of the reacting solution was deposited onto a TEM grid. The grids were immediately rinsed with anhydrous ethanol to terminate the reaction and remove residual soluble salts, followed by air-drying at RT before imaging.

An additional control experiment was conducted to evaluate HAP crystallization in an artificial saliva (AS). The reaction system contained 20 mM CaCl_2_, 3 mM K_2_HPO_4_, 150 mM NaCl, 0.77 mM NaF, and 10 mM Tris buffer at pH 8.5. To capture the morphological evolution of the precipitates over an extended duration, aliquots were sampled at designated times. For TEM characterization, 5 μL of the solution was deposited onto a TEM grid, followed by a brief rinse with anhydrous ethanol to remove excess salts. The grids were subsequently air-dried before analysis.

**1.8 High-speed Atomic Force Microscopy (HS-AFM)**

All dynamic assembly processes were monitored by HS-AFM using an SS-NEX instrument (RIBM, Japan) equipped with diamond-like-carbon (DLC)-tipped ultra-short cantilevers (NanoWorld USC-F1.2-k0.15-10). The cantilevers (7 μm × 2 μm × 80 nm; force constant: 0.15 N/m) were operated at a nominal resonance frequency of 1.2 MHz. This configuration minimizes hydrodynamic damping in liquid environments, enabling continuous imaging at 20 frames/s for real-time tracking of nanoparticle assembly. The enzymatic mineralization reaction mixture was injected into the AFM fluid cell, and sequential scanning was initiated immediately upon reaction commencement.

**1.9 Enamel Crystal Bundle Preparation**

Sound human enamel specimens were etched with 37% phosphoric acid for 20 s and then subjected to ultrasonic agitation in deionized water for 3 min. The enamel surface was gently scraped with a surgical scalpel, and the blade was rinsed in deionized water to transfer the dislodged material into suspension. The suspension containing enamel crystal bundles was collected, and a droplet was deposited onto a clean copper TEM grid and air-dried before imaging.

**1.10 Observation of Mineral Evolution in EMS without F⁻**

The evolution of CaP particles in the absence of F^-^ was monitored by TEM. The reaction system was identical to the EMS except that NaF was removed, containing 20 mM CaCl_2_, 2 μg/mL ALP, 3 mM β-GP, 150 mM NaCl, and 10 mM Tris buffer at pH 8.5. At designated time points (30 min, 2 h, 3 h, and 48 h), 5 μL of the reaction solution was dropped onto a TEM grid, gently rinsed with ethanol, and subsequently air-dried at room temperature before imaging. TEM, HRTEM, SAED, and EDS analyses were performed using a JEOL JEM-F200 microscope equipped with dual Super-EDS detectors at an accelerating voltage of 80 kV.

**1.11 Solid-state Nuclear Magnetic Resonance (SSNMR)**

Solid-state ^13^C cross-polarization magic-angle spinning (CP/MAS) NMR spectra were acquired on a Bruker Avance III HD 600 MHz spectrometer (Bruker BioSpin, Germany) equipped with a 3.2 mm MAS probe. To investigate the chemical evolution during mineralization, solid precipitates were harvested from the EMS reaction system at designated times. Each aliquot was centrifuged at 12,000 rpm for 10 min and subjected to three times washing with anhydrous ethanol to remove residual soluble components. The resulting solids were dried under vacuum at 70^o^C for 24 h before analysis. NMR measurements were performed at a magic-angle spinning frequency of 15 kHz. Data processing and spectral analysis were conducted using MestReNova software (version 14.0, Mestrelab Research, Spain).

**1.12 Sodium Dodecyl Sulfate-polyacrylamide Gel Electrophoresis (SDS-PAGE) Analysis of Cy5-ALP**

The conjugation efficiency and purity of the Cy5-labeled ALP (Cy5-ALP) were validated via SDS-PAGE analysis. Electrophoretic separation was performed alongside a pre-stained protein ladder (Biosharp BL712A). Following electrophoresis, the gels were first subjected to in-gel fluorescence imaging using a Typhoon FLA 9500 scanner (λ_ex_ = 646 nm, λ_em_ = 668 nm) to selectively detect the Cy5-fluorophore distribution. Subsequently, the same gels were processed with Coomassie Brilliant Blue staining to visualize the total protein profile. The spatial correlation between the fluorescence signal and the total protein bands confirmed the successful and high-purity conjugation of Cy5 to the ALP molecules.

**1.13 Cy5-ALP Activity Assay**

The enzymatic activity of Cy5-ALP was assessed by monitoring the hydrolysis of pNPP, a standard chromogenic substrate for phosphatases. The reaction was initiated by introducing Cy5-ALP (final concentration: 2 μg/mL) into an alkaline phosphatase buffer containing 3 mM pNPP. The liberation of pNP was quantified by measuring the absorbance at 405 nm using a microplate reader (Synergy H1, BioTek, USA). Absorbance data were recorded at 30-minute intervals, and the enzymatic activity was calculated based on a pNP standard curve, ensuring that the Cy5-conjugation process did not compromise the functional performance of the enzyme.

**1.14 High-resolution Liquid Chromatograph Mass Spectrometer (LC-MS)**

The enzymatic hydrolysis of Cy3-β-GP and its resulting cleavage products were characterized using an ultra-high performance liquid chromatography (UHPLC) system coupled with a triple time-of-flight mass spectrometer (TripleTOF 6600, SCIEX, USA). Chromatographic separation was optimized to resolve the labeled substrate from its hydrolyzed fragments, followed by high-resolution mass spectrometry to determine their precise molecular weights and fragmentation patterns. Data acquisition and spectral processing were conducted using Analyst TF software (Version 1.7.0, SCIEX), with molecular structural assignments based on accurate mass-to-charge (*m/z*) ratios.

**1.15 Characterization of Bulk HAP Crystals**

The phase composition of the samples was characterized by X-ray diffraction (XRD). The XRD spectra were acquired using a Rigaku Ultima IV diffractometer (Rigaku Corporation, Japan) with Cu Kα radiation (λ = 1.542 Å). Measurements were performed in continuous scan mode at 40 kV and 40 mA over a 2θ range of 10°-70°, with a step size of 0.02°.

The surface morphology and elemental composition of the samples were examined using SEM and EDS. Observations were carried out on a SEM (SU8010, Hitachi, Japan) equipped with an Oxford X-max80 EDS detector. Before imaging, all specimens were sputter-coated with a thin gold/palladium (Au/Pd) conductive layer for 90 s using an ion sputtering coater (MC1000, Hitachi, Japan) to improve surface conductivity. SEM imaging was performed at an accelerating voltage of 4 kV in secondary electron imaging mode.

**1.16 In Situ Contact Angle Measurements and Interfacial Energy Calculations**

Static water contact angles (WCA) were first recorded on the freshly prepared pristine crystal facets. Subsequently, the crystal slices were immersed in a 10 mM β-GP solution (10 mM Tris buffer, pH 8.5) at room temperature for 5 min to reach adsorption equilibrium, gently dipped and rinsed in UPW for 5 s to eliminate residual unadsorbed β-GP molecules, and lightly dried under a high-purity N_2_ stream prior to immediate WCA measurement. Following the adsorption measurement, an ALP reaction solution (1 mg/mL in 10 mM Tris buffer, pH 8.5) was applied in situ onto the β-GP-treated crystal surfaces and incubated for 5 min. The enzymatic reaction was then terminated by gently rinsing the surface with UPW for 5 s to wash off the enzyme and cleaved glycerol products, followed by N_2_ drying for the final WCA measurement. Contact angle measurements were conducted at 25 ± 1°C via the sessile drop method using an optical contact angle goniometer (Attension Theta Flex, Biolin Scientific, Finland). A micro-syringe was used to dispense a 2.0 μL droplet of UPW onto the target facet, and droplet profiles were captured within 1 s.

The solid-liquid interfacial energy (*γ_SL_*) between the crystal facet and the aqueous phase was quantitatively evaluated using the Kwok-Neumann equation of state (EOS):

$$\text{cos}\text{θ}\text{=-1+2}\sqrt{\frac{\text{γ}_{\text{S}}}{\text{γ}_{\text{L}}}}\exp\left[ \text{-β}\left( \text{γ}_{\text{L}}\text{-}\text{γ}_{\text{S}} \right)^{\text{2}} \right]$$

$$\text{γ}_{\text{SL}}\text{=}\text{γ}_{\text{S}}\text{-}\text{γ}_{\text{L}}\text{cos}\text{θ}$$

Where *θ* is the measured WCA, *γ_L_* = 72.8 mJ/m^2^ is the surface tension of pure water, β = 0.0001247 (m^2^/ mJ)^2^ is an empirical constant, and *γ_S_* represents the derived solid surface energy [S1].

**1.17 ALP Activity & Inhibition Assay**

The enzymatic activity of ALP and its inhibition by levamisole were evaluated using the chromogenic hydrolysis of pNPP to pNP. A standard calibration curve was established using pNP solutions (0.0, 0.2, 0.4, 0.6, 0.8, 1.0 mM) to correlate absorbance at 405 nm with product concentration. The reaction was performed in a 200 μL volume containing 3 mM pNPP in 10 mM Tris buffer (pH 8.5) and 2 μg/mL ALP, in both the presence and absence of 7.5 mM levamisole as a specific inhibitor. The mixture was incubated at 37^o^C, and the absorbance at 405 nm was recorded in real-time at 5-minute intervals for a total duration of 60 min using a Synergy H1 microplate reader (BioTek, USA).

**1.18 Effects of an ALP Inhibitor on the Assembly of the HAP Formed in EMS**

To investigate the enzymatic dependence of mineralization, HAP precipitates were harvested from the EMS in both the presence and absence of levamisole, a specific ALP inhibitor. In the inhibition group, levamisole was introduced after 2 h of reaction to a final concentration of 7.5 mM, followed by an additional 1 h of incubation. A corresponding experimental group underwent a continuous 3 h reaction without inhibitor intervention. The resulting minerals were characterized using TEM, high-resolution TEM (HRTEM), and scanning transmission electron microscopy (STEM) on a JEOL JEM-F200 microscope equipped with dual Super-EDS detectors (accelerating voltage: 80 kV). For thermogravimetric analysis (TGA), the HAP particles were isolated via centrifugation (10,000 rpm, 12 min), washed sequentially with ultrapure water (once) and ethanol (twice), and dried under vacuum overnight. TGA was performed under an air atmosphere using a Netzsch TG 209 F1 Libra analyzer (Germany). The thermal program comprised: an initial ramp from 30 to 150^o^C, an isothermal hold at 150^o^C for 30 min to remove adsorbed water, a second ramp to 400^o^C with a 30 min hold to decompose organic components, and a final heating stage to 800^o^C at a constant rate of 10^o^C/min.

**1.19 Cyclic Remineralization of Enamel**

Acid-etched enamel samples were immersed in the EMS at 37^o^C. The remineralization process consisted of three successive 2-day cycles, for a total of 6 days. At the end of each cycle, the medium was replenished with freshly prepared EMS to maintain optimal ion concentration and enzymatic activity for sustained mineral deposition. Following the completion of all cycles, the samples were ultrasonically cleaned in deionized water for 5 min to remove loosely attached precipitates and then air-dried before further characterization.

**1.20 Micro-scratch Adhesion Test**

The enamel specimens were remineralized in EMS for 2 days and subsequently subjected to micro-scratch testing using an Rtec SMT-5000 multifunctional tribometer (Rtec Instruments, San Jose, CA, USA). A conical diamond indenter with a cone angle of 90° and a tip radius of 50 μm was used. The test was conducted under a constant normal load of 0.5 N at a scratching velocity of 1.5 mm/min over a distance of 0.5 mm (Pre-Scratch-Post mode).

**1.21 SEM Characterization of GelMA Hydrogel**

The GelMA hydrogel used for EMS loading was prepared and photocrosslinked as described in Section 4.17 of the maintext. The crosslinked hydrogels were pre-frozen at -80°C and lyophilized for 48 h using a freeze-dryer (Scientz-100F, Ningbo Scientz Biotechnology Co., Ltd., Ningbo, China). Before imaging, the lyophilized hydrogel samples were sputter-coated with a thin Au/Pd conductive layer for 90 s using an ion sputteringcoater (MC1000, Hitachi, Japan). The microstructure of the samples were examined by SEM (SU8010, Hitachi, Japan) in secondary electron imaging mode at an accelerating voltage of 4 kV.

**1.22 Biosafety Test**

L929 fibroblast cells (RRID: CVCL_0462) were seeded into 24-well plates at a density of 1 × 10^4^ and incubated for 24 h to allow for initial adherence. To evaluate the cytocompatibility of the scaffolds, GelMA hydrogels with or without incorporated EMS were loaded into Transwell inserts (5 μm pore size, Labselect, China) and co-cultured with the underlying L929 cells for 1, 3, and 5 days. At each time point, cell viability was assessed using the cell counting kit-8 (CCK-8) assay (GK10001, GlpBio, USA). Briefly, the culture medium was replaced with fresh medium containing 10% (v/v) CCK-8 reagent, followed by a 2 h incubation at 37^o^C. The absorbance at 450 nm was recorded using a Synergy H1 microplate reader (BioTek, USA), with six biological replicates (N = 6) per group. For morphological visualization, cells were fixed with 4% paraformaldehyde (P0099, Beyotime, China) for 20 min, permeabilized with Immunostaining Permeabilization Solution (P0096, Beyotime) for 5 min, and blocked with Immunostaining Quick Block (P0260, Beyotime) for 10 min. The cytoskeleton and nuclei were sequentially stained with Actin-Tracker Red-555 (C2203S, Beyotime) and DAPI staining solution (C1006, Beyotime), respectively. Fluorescence signals were captured using a confocal laser scanning microscope (CLSM; Nikon A1, Tokyo, Japan) to assess cell spreading and attachment.

**Supplementary Figures**

Figure S1. **Spatiotemporal distribution of alkaline phosphatase (ALP) in developing teeth.** (A) Representative immunofluorescence image of a mouse incisor tooth germ during the developmental stage. Nuclei are counterstained with DAPI (blue), while ALP (red) indicates its active expression during odontogenesis. Scale bar: 10 μm. (B) Immunofluorescence staining of a mature molar hard tissue section, confirming the postnatal retention of ALP (red) within the tooth enamel. Scale bar: 200 μm.

Figure S2. **Characterization of ALP-mediated β-GP hydrolysis and mineralization initiation.** (A) Liquid-state ³¹P NMR spectra show that the characteristic peak of β-GP appears at 0.5 ppm, while the peak for inorganic phosphate (PO_4_^3-^) appears at 4.2 ppm. Upon the addition of ALP to the β-GP solution, the emergence of the phosphate peak, accompanied by the disappearance of the β-GP signal, successfully demonstrated the enzymatic hydrolysis of β-GP. (B) Digital photographs depicting the macroscale transition of the reaction system. The clear solution containing β-GP and calcium ions undergoes rapid opacification upon ALP introduction, indicating the onset of calcium phosphate nucleation and precipitate formation. (C) Kinetic profile of total PO_4_^3-^ generation (including free and mineral-bound phosphate) during the enzymatic hydrolysis of β-GP. The hydrolysis rate exhibits a biphasic character, with a rapid initial phase within the first 6 h, followed by a plateauing phase. Notably, the hydrolysis remains incomplete after 48 h, suggesting a sustained and controlled ion release mechanism.

Figure S3. Interfacial association between CaP clusters and **β-GP** characterized by AFM-IR. Representative AFM height images displaying (A) synthesized CaP clusters and (C) a control gold-coated mica substrate. (B, D) Corresponding AFM-IR absorption maps (peak-to-peak amplitude) acquired at 1130 cm^-1^, which is assigned to the asymmetric P-O-C stretching vibration of β-GP, revealing the spatial distribution of the organic matter on the mineral phase. (E) FTIR spectrum of β-GP shows characteristic peaks at 1127 and 1057 cm^-1^, which are attributed to P-O-C stretching vibrations. (F) Representative nano-IR point spectra obtained from CaP clusters (red) and the gold-coated mica control (black). The presence of a distinct absorption band at 1130 cm^-1^ specifically on the clusters, and its absence on the substrate, confirms the localized enrichment and direct association of β-GP with the CaP clusters. Scale bars: 200 nm (A-D).

Figure S4. Raman spectroscopic analysis of the EMS mineralization process. The Raman spectrum of pristine β-GP displays characteristic peaks at 980 cm^-1^ and 1058 cm^-1^, corresponding to the symmetric stretching modes of the P-O and P=O bonds, respectively. Analysis of CaP particles harvested at various intervals reveals a peak at 953 cm^-1^, assigned to the symmetric stretching of PO_4_^3-^ in the calcium phosphate mineral. Notably, the intensity of the peak at 953 cm^-1^ significantly increases from 2 min to 1 h, confirming the rapid progression of enzymatically mediated mineralization. Concurrently, the β-GP peaks persist but exhibit significant blue shifts: the symmetric stretching of P-O shifted from 980 cm^-1^ to 1002 cm^-1^ (2 min), 1000 cm^-1^ (30 min), and 994 cm^-1^ (1 h), while the symmetric stretching of P=O shifted from 1058 cm^-1^ to 1069 cm^-1^ (2 min and 30 min), and 1077 cm^-1^ (1 h). These progressive blue shifts, combined with the observed signal attenuation, indicate strong interfacial interactions and localized chemical environment changes as β-GP integrates with and is hydrolyzed by the growing mineral phase.

Figure S5. **Evolution of CaP mineral in the absence of ALP.** Representative TEM images illustrating the dynamic evolution of CaP particles over time. (A) Initial nucleation of small, discrete CaP clusters was observed after 2 min of incubation. (B) No significant morphological transition or crystalline maturation was evident after 1 h, with the clusters maintaining their initial state. (C) Upon extending the incubation to 3 h, the primary clusters underwent random aggregation into larger spherical ACP particles. Notably, the stabilization of CaP particles by adsorbed β-GP inhibited their further transformation, with no spindle-shaped hierarchical structures or rod-like HAP crystals being detected. These results underscore that the enzymatic hydrolysis of β-GP is a prerequisite for triggering the transition from amorphous precursors to organized crystalline assemblies in the EMS. The inset displays the corresponding SAED pattern, in which the absence of discernible diffraction spots or rings confirms the amorphous state of the particle. (D) HRTEM image of the boxed region in (C) reveals no observable lattice fringes, further verifying the lack of long-range crystalline order. Scale: 100 nm (A, B); 200 nm (C); 5 nm (D); 2 nm^-1^ for the inset in (C).

Figure S6. **Elemental mapping of the evolving CaP particles.** Elemental maps confirm the consistent co-localization of calcium (Ca) and phosphorus (P), which constitute the inorganic mineral phase. The overlapping signal of nitrogen (N) originates from the amine groups of the ALP enzyme, confirming the integration of the organic mediator within the mineral across all developmental stages. Representative elemental distributions are shown for particles harvested at: (A) 2 min, (B) 1 h, (C) 2 h, (D) 3 h, (E) 24 h, and (F) 48 h. Scale bars: 50 nm (A); 200 nm (B, F); 500 nm (C); 20 nm (D); 100 nm (E).

Figure S7. Real-time HS-AFM visualization of the *in situ* self-assembly of CaP particles into spindle-shaped architectures within the EMS. (A-E) Sequential HS-AFM height images captured in aqueous solution, showing the dynamic assembly of CaP particles. Initial CaP particles are observed aggregating into a nascent spindle-shaped structure (highlighted in orange) in (A), followed by sustained growth through the continuous integration of CaP particles (likely nanorods) in (B) and (C). The rapid disappearance of primary particles (within 1 s) coincides with the volume expansion of adjacent spindle-shaped mineral, suggesting a particle-mediated attachment mechanism. These real-time observations provide direct evidence that CaP growth within the EMS is driven by the hierarchical assembly of primary building blocks rather than classical ion-mediated growth. Scale bar: 50 nm (consistent across all panels). Time stamps denote minutes and seconds from the initial solution mixing.

Figure S8. **Ultrastructural characterization of natural enamel rods.** A representative TEM micrograph displays a spindle-shaped assembly of HAP crystallites exfoliated from the enamel surface. The bundle is composed of highly co-aligned, rod-like nanocrystals, illustrating the sophisticated hierarchical architecture and organized packing characteristic of native mature enamel. Scale bars: 200 nm.

Figure S9. **Morphological evolution and phase transformation of CaP minerals in AS.** Representative TEM micrographs illustrating the time-dependent crystallization kinetics of CaP in a simulated physiological environment. (A) Formation and aggregation of spherical ACP nanoparticles after 30 min of reaction. (B) Initiation of the amorphous-to-crystalline transition after 1 h, with nascent HAP nanocrystals (indicated by arrows) emerging on the surfaces of the ACP precursors. (C) Development of discrete, rod-like HAP crystals after an extended incubation of 48 h, reflecting slow maturation and grain growth in the absence of enzymatic mediation. (D) HRTEM image of HAP crystals. The identified lattice fringes with a d-spacing of 0.34 nm correspond to the (002) crystallographic planes of HAP. Scale bars: 100 nm (A-C); 5 nm (D); 2 nm^-1^ (SAED in (C)).

Figure S10. **TEM characterization of mineral formation in EMS without F^-^.** (a) After 30 min of mineralization, a continuous layer of ACP was formed. (b) After 2 h, crystalline HAP began to emerge. (c) After 3 h, plate-like HAP crystals became clearly visible. Inset: SAED pattern displaying diffraction rings corresponding to the (211) planes of HAP. (d) After 48 h, highly crystalline plate-like HAP crystals had formed. Inset: SAED pattern showing distinct diffraction rings of HAP. (e) HRTEM image of the region indicated by the white square in (d), revealing clear lattice fringes with a d-spacing of 0.34 nm corresponding to the (002) plane of HAP. (f) STEM image and corresponding EDS elemental mapping confirming the co-localization of calcium (Ca), phosphorus (P), and oxygen (O). Scale bars: 500 nm (a, b); 100 nm (c); 200 nm (d, f); 5 nm (e). SAED scale bars: 5 nm^-1^ (a-d).

Figure S11. **Solid-state ^13^C NMR analysis of the CaP minerals.** Time-resolved Solid-state ^13^C NMR spectra were employed to track the molecular fate of the β-GP within the evolving CaP mineral collected at different time points. The spectra exhibit a progressive, time-dependent attenuation of the characteristic β-GP peaks at 63 ppm and 75 ppm, corresponding to the methylene (C-OH) and methine (C-O-PO_3_) carbons, respectively. This reduction in signal intensity provides direct molecular-level evidence of the ALP-mediated hydrolysis of β-GP at the mineral interface, confirming that β-GP is actively consumed to drive the sustained growth and maturation of the HAP crystals.

Figure S12. **Synthesis, characterization, and functional validation of fluorophore-conjugated ALP and β-GP.** (A) SDS-PAGE analysis with Coomassie Brilliant Blue staining. Lane 1: Protein molecular weight marker (kDa; Biosharp BL712A). Lane 2: ALP. Lane 3: Cy5-conjugated ALP. They migrate at approximately 70 kDa, and the retention of the protein band in Lane 3 confirms that the polypeptide backbone remains intact after Cy5 labeling. (B) In-gel fluorescence imaging (Cy5 channel; λ_ex_ = 646 nm, λ_em_ = 668 nm). Specific fluorescence is detected exclusively in Lane 3, confirming the successful covalent conjugation of Cy5 to ALP. The absence of signal in Lanes 1 and 2 demonstrates the lack of non-specific fluorescence. (C) Catalytic activity assay of Cy5-ALP: The hydrolysis of pNPP by Cy5-ALP confirms that the fluorophore conjugation does not compromise the robust catalytic activity of the enzyme toward monophosphoester substrates. (D) UHPLC-MS analysis of Cy3-β-GP and its enzymatic hydrolysate. The molecular ion peak of Cy3-β-GP is identified at *m/z* 612.7, while the peak at *m/z* 457.3 corresponds to the major fragment ion generated upon hydrolysis. These results confirm that Cy3-β-GP was successfully hydrolyzed by Cy5-ALP. (E) Chemical structure of the synthetic substrate Cy3-β-glycerophosphate (Cy3-β-GP; Mw = 611.7 g/mol). (F) Chemical structure showing the hydrolyzed fragments. The fragment detected by MS (457.3 g/mol) is marked within the dashed box.

Figure S13. **Morphological and crystallographic characterization of millimeter-sized HAP crystals.** (A) Optical photograph displaying the macroscale dimensions of the HAP crystals. (B) XRD patterns demonstrating highly oriented growth. The surface-parallel diffraction reveals a series of sharp peaks at *2θ* = 10.8°, 21.8°, 32.9°, and 44.4°, which are indexed to the (100), (200), (300), and (400) reflections, respectively. The XRD pattern of the cross-section exhibits a predominant peak at *2θ* = 25.9°, corresponding to the (002) facet. All diffraction peaks align with the standard HAP (JCPDS No. 09-0432), confirming phase purity and a preferred orientation along the *c*-axis. (C) SEM image showing the surface morphology. Scale bar: 20 μm. (D-F) Corresponding EDS elemental mapping confirming the uniform distribution and co-localization of (D) calcium (Ca), (E) phosphorus (P), and (F) oxygen (O) across the crystal surface. Scale bars: 20 μm.

Figure S14. **Radial distribution function (RDF) analysis of** **β-GP on the HAP surfaces.** Radial distribution function, *g(r)*, of β-GP phosphate oxygen atoms relative to surface Ca²⁺ ions on the HAP (100) and (002) facets. The red and blue curves represent the (100) and (002) surfaces, respectively. A prominent coordination peak at *r* ≈ 2.38 Å characterizes the direct coordination between the phosphate oxygens and surface calcium sites. The peak intensity for the (100) surface (3.93) is substantially higher than that for the (002) surface (1.46), demonstrating a more probable binding affinity of the β-GP phosphate groups towards the Ca^2+^ sites on the (100) facet.

**Figure S15. WCA measurements on HAP crystal facets.** Representative optical photographs of water droplets on the HAP (A) (100) and (B) (002) facets at three sequential stages: pristine state, after β-GP adsorption, and following in situ ALP enzymatic hydrolysis.

Figure S16. Disruption of hierarchical assembly of HAP nanorods via inhibition of β-GP hydrolysis. (A) TEM image of HAP crystals formed within the EMS (3 h total reaction), where the ALP inhibitor levamisole was introduced at 2 h. The HAP crystals remain randomly dispersed, failing to organize into the characteristic spindle-shaped aggregates. The individual HAP nanorods measure 24.85 ± 6.69 nm in length and 6.18 ± 0.93 nm in width (aspect ratio approximately 4.0), consistent with the dimensions of HAP nanorods observed in the EMS. Inset: SAED pattern displaying diffraction rings/spots corresponding to the (002), (211), and (004) crystallographic planes of HAP. Scale bar, 200 nm, 5 nm^-1^ for SAED. (B) HRTEM image of the region marked by the white square in (A), revealing well-defined lattice fringes with interplanar spacings indexed to the (002) and (211) planes of HAP. Scale bar, 5 nm. (C) STEM imaging and corresponding elemental mapping of the HAP crystals revealed the distribution of its constituent elements: Calcium (Ca), Phosphorus (P), and Oxygen (O). Scale bar, 100 nm. (D) ALP inhibition assay showing that 7.5 mM levamisole achieves an 84% inhibition rate of pNPP hydrolysis within 1 h, indicating potent suppression of the β-GP hydrolysis, hinting that levamisole can also be effective in inhibiting the ALP-mediated hydrolysis of β-GP in the EMS. (E-F) TGA and corresponding derivative thermogravimetry (DTG) curves of HAP nanorods obtained from the EMS after a 3-hour reaction: (E) EMS without levamisole, and (F) EMS with the ALP inhibitor levamisole (7.5 mM) introduced at 2 h. (G) Quantitative analysis of adsorbed organic content (primarily β-GP) on the HAP surface derived from TGA: 71.82 ± 1.13 μg/mg (without inhibitor) versus 77.07 ± 1.36 μg/mg (with levamisole). The higher residual organic content in the inhibited group confirms that levamisole suppresses the hydrolysis of β-GP molecules adsorbed on the HAP surfaces, thereby inhibiting the hierarchical assembly process.

Figure S17. **Optical profilometry analysis of enamel remineralization within the EMS.** (A) Representative optical profilometry image depicting the topographical boundary between the remineralized enamel (green) and the native enamel (blue). Scale bar, 20 μm. (B) Cross-sectional height profile extracted along the black dashed line indicated in (A), quantifying a repaired layer thickness of approximately 1.92 μm. Horizontal dashed lines represent the mean elevation of the respective surfaces.

Figure S18. Restoration of enamel across distinct microstructural orientations. (A) Acid-etched enamel (AE), the outermost layer of the tooth, exhibits a honeycomb-like structure. (B) Optical image of the whole sound enamel. (C) After remineralization in EMS, enamel was repaired, replicating the original honeycomb-like structure. (D) AE displayed parallelly arranged enamel rods. (E) Optical image of enamel showing a longitudinal cross-section. (F) Newly formed HAP layer on enamel after EMS remineralization, showing an organized structure similar to native enamel. Scale bars: 5 μm (A, C, D, F); 1 μm (Insets).

Figure S19. Escalation of remineralization thickness via a cyclic treatment strategy. The thickness of the regenerated mineral layer on the enamel surface is significantly enhanced through cyclic remineralization within the EMS. As the number of remineralization cycles increases from one to three, the total thickness of the repaired layer increases proportionally from 2 μm (A) to 6 μm (B), demonstrating the scalability of the EMS-based restoration process. Scale bar: 500 nm (A); 1 μm (B).

Figure S20. Comprehensive mechanical characterization of enamel samples. (A) Representative load-displacement curves obtained via quasi-static nanoindentation. (B) Representative oscillatory load-displacement profiles acquired in dynamic mechanical analysis (DMA) mode. These data were utilized to calculate the storage modulus and damping coefficient. (C) Coefficient of friction (*μ*) measured by tribological testing, showing its evolution over time.

Figure S21. **SEM images of the scratch track on EMS-remineralized enamel.** Representative SEM morphology of the scratch track on repaired enamel, demonstrating intact scratch edges with no evidence of interfacial delamination, cracking, or spallation. Scale bar 100 μm.

Figure S22. Microstructure of the GelMA hydrogel used for EMS loading. (A) Representative SEM image of the GelMA hydrogel. Scale bar: 100 μm. (B) Quantitative analysis of the pore size distribution based on SEM images. The mean pore size was 126.4 ± 19.1 μm (mean ± SD).

Figure S23. Cytocompatibility assessment of the EMS-hydrogel. (A) CCK-8 proliferation assay demonstrating that the EMS-hydrogel supports L929 fibroblast growth with no significant difference compared to the control group over a 5-day culture period (N = 6 per group). (B) Representative confocal immunofluorescence micrographs of L929 cells cultured on the EMS hydrogel. The cells exhibit well-spread morphologies and organized cytoskeletal architectures (F-actin, red; DAPI, blue) comparable to those on the control substrate, indicating healthy cellular attachment and spreading. These findings collectively confirm the excellent biocompatibility and non-cytotoxic nature of the EMS platform. Scale bar: 20 μm.

Figure S24. SEM characterization of CaP mineral structure within the EMS hydrogel. (A) Low-magnification SEM image displaying the widespread formation of CaP mineral after 48 h of incubation. (B) High-magnification view of the region demarcated by the white dashed box in (A), revealing the initial nucleation and growth of discrete HAP nanorods. (C) High-magnification SEM image of the yellow dashed area in (A), illustrating the hierarchical assembly of nanorods into larger, spindle-shaped assemblies. These observations confirm that the multi-stage assembly of HAP nanorods, a hallmark of the EMS process, is consistently maintained within the hydrogel matrix. Scale bars: 2 μm (A), 500 nm (B), 1 μm (C).

Figure S25. Enamel remineralization facilitated by the EMS hydrogel. (A-D) Schematic illustration of the experimental procedure for enamel remineralization: (A) Acid-etched enamel. (B) Dropping the EMS-GelMA precursor solution onto the etched enamel surface. (C) *In situ* photopolymerization (light-curing) to induce rapid gelation, stabilizing the mineralizing environment on the enamel surface. (D) Immersion of the treated enamel sample in artificial saliva (AS) for remineralization. (E-G) Representative SEM images of the control group (direct immersion of etched enamel in AS without EMS hydrogel): High-magnification surface and cross-sectional views reveal disordered, plate-like CaP precipitates that fail to replicate the native organized architecture. (H-J) Representative SEM images of the control group with AS containing F⁻: rod-like HAP crystals formed but grew in a disordered, random orientation. (K-M) Representative SEM images of the EMS-hydrogel-treated enamel: The remineralized surface exhibits a characteristic fish-scale-like microstructure that is similar to that of natural enamel. Notably, the cross-sectional analysis confirms the formation of an organized HAP layer with a preferred crystallographic orientation, maintaining structural continuity with the underlying native enamel rods. Scale bars: 5 μm (E, H, K); 1 μm (F, G, I, J, L, M).

**3. Table S1**

Experimental WCA *(θ)* and calculated solid-liquid interfacial energies (*γ_SL_*) on HAP (100) and (002) crystal facets across different treatment stages.

| Crystal surface | Treatment Stage | WCA (*θ*,degrees) | *γ_SL_* (mJ/m^2^) | Recovery of *γ_SL_* (%) |
| --- | --- | --- | --- | --- |
| (100) | Pristine | 64.61 ± 1.27° | 13.81 | – |
|  | + β-GP | 48.25 ± 0.60° | 6.29 | – |
|  | + ALP | 54.44 ± 1.68° | 8.82 | 33.6% |
| (002) | Pristine | 74.75 ± 1.21° | 19.61 | – |
|  | + β-GP | 56.10 ± 1.30° | 9.58 | – |
|  | + ALP | 72.57 ± 0.91° | 18.32 | 87.1% |

Note: The recovery rate of solid-liquid interfacial energy is defined as $Recovery =\frac{\gamma_{SL}^{\left( \text{ALP} \right)}-\gamma_{SL}^{\left( \beta\text{-GP} \right)}}{\gamma_{SL}^{\left( \text{Pristine} \right)}-\gamma_{SL}^{\left( \beta\text{-GP} \right)}}\text{×100\%.}$

**4. Supplementary Text 1**

The interfacial energies of the (100) and (002) facets of HAP crystals were calculated directly from the WCA measurements. Pristine HAP exhibits an intrinsic interfacial energy anisotropy, with the (002) facet showing a significantly higher *γ_SL_* (19.61 mJ/m²) than the (100) facet (*γ_SL_* = 13.81 mJ/m²). Accordingly, the intrinsic anisotropy ratio $\gamma_{SL}^{\left( \text{002} \right)}\text{/}\gamma_{SL}^{\left( 100 \right)}\text{≈}\text{ }\text{1.42}$, in close agreement with the value of ~1.5 predicted by our MD simulations. Upon β-GP adsorption *γ_SL_* decreased by more than 50% on both facets, which from 13.81 reduce to 6.29 mJ/m^2^ on (100) facet and from 19.61 reduce to 9.58 mJ/m^2^ on (002) facet. Consistently, the lower WCA observed on the (100) facet (48.25°) than on the (002) facet (56.10°) experimentally supports the higher molecular packing density and stronger interfacial coordination predicted by the MD calculations (Figure S15 and Table S1).

Notably, subsequent in situ ALP-mediated hydrolysis induced a facet-selective recovery of interfacial energy. On the (002) facet, the more weakly bound β-GP was readily hydrolyzed, resulting in an 87.1% recovery of *γ_SL_* to 18.32 mJ/m^2^ and nearly complete re-exposure of the intrinsically high-energy surface. In contrast, the strongly anchored β-GP layer on the (100) facet likely imposed steric constraints on the accessibility of phosphate ester bonds, leading to only a 33.6% recovery of *γ_SL_* to 8.82 mJ/m^2^. This differential enzymatic turnover increased the interfacial-energy anisotropy to $\gamma_{SL}^{\left( \text{002} \right)}\text{/}\gamma_{SL}^{\left( 100 \right)}\text{≈}\text{ }\text{2}\text{.}\text{08}$, closely matching the maximum ratio of ~2.1 predicted by the MD simulations. These results indicate that facet-selective enzymatic hydrolysis dynamically amplifies the interfacial-energy contrast between the two HAP facets, thereby providing a thermodynamic driving force favorable for *c*-axis-oriented attachment.

**5. Supplementary Reference**

[S1]. D. Y. Kwok, A. W. Neumann, Contact angle measurement and contact angle interpretation, *Advances in Colloid and Interface Science*, 1999, 81(3): 167–249.
